# Old Passengers as New Drivers: Chromosomal Passenger Proteins Engage in Translesion Synthesis

**DOI:** 10.3390/cells13211804

**Published:** 2024-10-31

**Authors:** Katharina Falke, Elisabeth Schröder, Stefanie Mosel, Cansu N. Yürük, Sophie Feldmann, Désirée Gül, Paul Stahl, Roland H. Stauber, Shirley K. Knauer

**Affiliations:** 1 Institute for Molecular Biology II, Center of Medical Biotechnology (ZMB), University of Duisburg-Essen, Universitätsstrasse 5, 45141 Essen, Germany; 2 Molecular and Cellular Oncology, University Medical Center Mainz, Langenbeckstrasse 1, 55101 Mainz, Germany; guel@uni-mainz.de (D.G.); rstauber@uni-mainz.de (R.H.S.)

**Keywords:** Survivin, Aurora B kinase, DNA damage response, replication, translesion synthesis

## Abstract

Survivin is known for its dual biological role in apoptosis inhibition and mitotic progression. In addition to its being part of the chromosomal passenger complex (CPC), recent findings suggest additional roles for Survivin in the DNA damage response, further contributing to therapy resistance. In this study, we investigated the role of Survivin and the CPC proteins in the cellular response to irradiation with a focus on DNA replication processes. As is known, ionizing radiation leads to an increased expression of Survivin and its accumulation in nuclear foci, which we now know to be specifically localized to centromeric heterochromatin. The depletion of Survivin and Aurora B increases the DNA damage marker γH2AX, indicative of an impaired repair capacity. The presence of Survivin and the CPC in nuclear foci that we already identified during the S phase co-localize with the proliferating cell nuclear antigen (PCNA), further implying a potential role during replication. Indeed, Survivin knockdown reduced replication fork speed as assessed via DNA fiber assays. Mechanistically, we identified a PIP-box motif in INCENP mediating the interaction with PCNA to assist in managing damage-induced replication stress. Survivin depletion forces cells to undergo unphysiological genome replication via mitotic DNA synthesis (MiDAS), resulting in chromosome breaks. Finally, we revealed that Aurora B kinase liberates Pol η by phosphorylating polymerase delta-interacting protein 2 (POLDIP2) to resume the replication of damaged sites via translesion synthesis. In this study, we assigned a direct function to the CPC in the transition from stalled replication forks to translesion synthesis, further emphasizing the ubiquitous overexpression of Survivin particularly in tumors. This study, for the first time, assigns a direct function to the chromosomal passenger complex, CPC, including Survivin, Aurora B kinase, Borealin, and INCENP, in the transition from stalled replication forks (involving PCNA binding) to translesion synthesis (liberating Pol η by phosphorylating POLDIP2), and thus in maintaining genomic integrity.

## 1. Introduction

Survivin, also known as a baculoviral inhibitor of apoptosis protein (IAP) repeat-containing protein 5 (BIRC5), is a small 16.5 kDa protein with 142 amino acids, which is essential for key cellular processes including apoptosis and mitosis [1]. The N-terminal baculoviral IAP repeat (BIR) domain characterizes Survivin as a member of the IAP family, interacting with pro-caspase-9 and resulting in the suppression of apoptosis. In contrast, the C-terminal α-helical domain (aa 98–142) of Survivin forms a three-helix bundle with Borealin (aa 15–79) and INCENP (aa 1–49), constituting the localization module of the chromosomal passenger complex (CPC), which is essential for chromosome alignment and segregation during mitosis [2]. Since the enzymatic component of the CPC, Aurora B kinase, is mainly regulated via its dynamic localization, Survivin fulfills a key regulatory function within the CPC by targeting the complex to Histone H3 and, finally, the centromeres. This enables Aurora B to phosphorylate a number of proteins, ultimately ensuring that the chromosomes are aligned properly before they are segregated at anaphase [1]. Importantly, Survivin harbors no enzymatic activity on its own but instead seemingly fulfills its physiological tasks by associating with other proteins as an adapter protein, merely interacting with or shuttling its partners to their intracellular destinations. This makes Survivin inherently difficult to target using small molecules or other traditional drug modalities because of the lack of suitable binding pockets and the need to block enlarged surface areas. Nevertheless, Survivin is considered an attractive therapeutic target. Its expression is critical for embryonic development and is mostly absent in terminally differentiated adult tissues. However, it has been shown to be abundantly upregulated in all cancer entities investigated, and it is the fourth most upregulated mRNA in cancer cells [3]. The increased expression of Survivin in malignancies is associated with high rates of tumor recurrence, abbreviated overall survival, and resistance to chemo- and radiotherapy.

Notably, recent studies suggest an additional role for Survivin, complementing its functions in apoptosis and mitosis. The overexpression of Survivin in cancer cell lines results in an increased resistance to radiation and an augmented DNA repair capacity [4,5]. Survivin interacts with several DNA repair factors, suggesting that it may directly participate in the DNA damage response (DDR) [6]. However, the exact molecular mechanism through which Survivin may boost DNA repair processes has not yet been elucidated.

Genome instability plays a significant role in the development and progression of cancer, with replication stress being a key factor driving this process [7]. DNA replication is an elaborate process involving the coordinated initiation of thousands of replicons and the accurate copying of the entire genome [8]. DNA synthesis must be coordinated with cell-cycle progression, transcription, DNA repair, and other cellular processes. Likewise, replication fork progression, which crucially depends on fork velocity, is tightly regulated [9]. As a direct consequence, the distance between single replication origins is linked to fork velocity, possibly because short inter-origin distances enhance torsional stress, which could, in turn, slow down fork progression. The progression of replication forks is occasionally hindered due to obstacles such as secondary DNA structures, repetitive sequences, certain protein–DNA complexes, bulky DNA lesions, and interstrand crosslinks, which are all referred to as DNA lesions. The stalling of replication forks constitutes a dangerous incident because incomplete chromosome duplication results in mitotic catastrophe, complex chromosomal rearrangements, and even cell death [10,11]. As such, replication forks are particularly fragile structures that need to be stabilized when stalled to prevent their collapse and, thus, the dissociation of the replisome, finally resulting in the generation of double-strand breaks (DSBs).

Cells have evolved several lesion-bypass mechanisms that can temporarily deal with DNA damage during replication, known as DNA damage tolerance (DTT) [10,11]. Template switching (TS) allows temporary changes in the stalled nascent strand to the newly synthesized, undamaged sister strand for replication on the lesion. Another DDT mechanism is translesion synthesis (TLS), which enables replication machinery to replicate past lesions by replacing regular replicative with specialized TLS polymerases, such as Pol η/ι/κ/ζ and REV1 [12]. The substitution of polymerases is initiated via the mono-ubiquitination of the proliferating cell nuclear antigen (PCNA), which is promoted via TLS polymerases themselves by serving as a scaffold, separate from their polymerization activity. Mono-ubiquitinated (Ub-) PCNA is poly-ubiquitinated, which leads to the initiation of TS [13].

As evidence that Survivin could be directly or indirectly involved in DNA repair is increasing, and the underlying mechanistic details remain unclear, we aimed to scrutinize its role in cells’ response to DNA damage induction at the molecular level. Consequently, cells’ response to ionizing radiation was assessed through protein expression, depletion, and co-localization studies relying on immunofluorescence staining and confocal microscopy. The underlying mechanisms were further elucidated via DNA fiber and proximity ligation assays. Furthermore, analyses of chromosome breaks were used to study mitotic DNA synthesis, and the mutagenesis mapping of protein interaction partners revealed the molecular link to translesion synthesis.

## 2. Materials and Methods

### 2.1. Cell Culture

Eukaryotic cells were cultivated in a humidified incubator at 37 °C, 5% CO_2_, and 90% relative humidity. Human HeLa (ATCC: CRM-CCL-2), U2OS (ATCC: HTB-96), A431 (ATCC: CRL-1555), WI-38 (ATCC: CCL-75), 293T (ATCC: CRL-3216), and murine NIH3T3 (ATCC: CRL-1658) cells were grown in Dulbecco’s Modified Eagle’s medium (DMEM) supplemented with 10% (*v*/*v*) fetal calf serum (FCS), 2 mM of L-Glutamine, and 1% (*v*/*v*) Antibiotic-Antimicotic (Gibco/Thermo Fisher Scientific, Dreieich, Germany). The stably transfected A431 Survivin-GFP cell line has been described previously and was maintained in a medium additionally containing 800 μg/mL of Geniticin (G418) (Gibco/Thermo Fisher Scientific, Dreieich, Germany) [14].

### 2.2. Plasmids

The eukaryotic expression constructs pc3-Survivin-GFP [15] and pc3-Survivin-HA [16] have been previously reported. The plasmid encoding human Survivin-tdTomato was constructed via polymerase chain reaction (PCR) amplification of the fluorescent tag from the tdTomato plasmid as a template [17] using appropriate primers (Eurofins, Ebersberg, Germany) containing NheI/EcoRI-restriction sites. The PCR product was used to exchange the GFP-Tag in pc3-Survivin-GFP with tdTomato, as previously described [15]. Plasmids pc3-Aurora B-HA and pc3-Borealin-HA were constructed through the PCR amplification of Aurora B and Borealin as templates [15] using appropriate primers containing NheI/EcoRI-restriction sites [15]. The PCR products were used to exchange the ORF of Survivin in pc3-Survivin-HA with Aurora B and Borealin, as previously described [15]. Plasmids encoding myc- or FLAG-tagged CPC members were likewise constructed by cloning the respective PCR-amplified ORF of Survivin, Aurora B, and Borealin containing NheI/EcoRI-restriction sites into a pc3-myc or a pc3-FLAG vector, as previously described [15]. The constructs peGFP-N3_HP1α and pcDNA3.1-myc-INCENP were provided courtesy of Prof. H. Meyer, University of Duisburg-Essen, Germany. To generate the INCENP-PIP box mutant (^853^**Q**AI**I**HQ**YY**^860^ → ^853^**A**AI**A**HQ**AA**^860^), the pcDNA3.1-myc-INCENP plasmid was used as a template for site-directed mutagenesis with PCR primers bearing Gln853, Ile856, Tyr859, and Tyr860 to alanine mutations resulting in the construct pcDNA3.1-myc-INCENP-PIPmut. The Q5^®^ Site-directed mutagenesis kit (New England BioLabs, Frankfurt a. Main, Germany) was used according to the manufacturer’s instructions. The construct pENeGFP-PCNA coding for GFP-PCNA was provided courtesy of Prof. C. Cardoso, Technical University Darmstadt, Germany [18]. pEN-HA-PCNA encoding HA-tagged PCNA was constructed by exchanging the ORF of GFP with an HA-tag generated through oligo annealing with synthetic primers containing BamHI/SacII-restriction sites. Plasmid pIRES-FLAG-POLH encoding FLAG-tagged polymerase Eta/Pol η was provided courtesy of Prof. C. Masutani, Nagoya University, Tokyo, Japan [19]. pcDNA3.1(+) (Life Technologies/Thermo Fisher Scientific, Dreieich, Germany) was used as an empty vector for transfection. A tGFP-tagged human POLDIP2 ORF clone (RG200053, Origene, Herford, Germany) was used to generate the POLDIP2 mutant T216E using the NEBaseChanger kit (New England BioLabs, Frankfurt am Main, Germany) according to the manufacturer’s instructions. Both POLDIP2 variants, wildtype and mutant, were successively cloned into the eukaryotic expression vector pEGFP-N1 (6085-1, Clontech/TaKaRa Bio, Saint-Germain-en-Laye, France) via a Gibson assembly of fragments using the NEBuilder HiFi DNA Assembly master mix (New England BioLabs, Frankfurt am Main, Germany) according to the manufacturer’s protocol [20].

### 2.3. Transfection

The transient transfection of eukaryotic cells with plasmid DNA was achieved via lipofection using Lipofectamine2000 and Opti-MEM (Invitrogen/Thermo Fisher Scientific, Dreieich, Germany) for the transfection of HeLa, A431, U2OS, and WI-38 cells or polyethylenimine (PEI) from Sigma-Aldrich/Merck KGaA (Darmstadt, Germany) for the transfection of 293T cells. Hereinafter, the transfection of cells in 3 cm dishes and a volume of 2 mL medium is described, and the noted volumes were adjusted accordingly for other dishes, plates, and flasks. For Lipofectamine2000-mediated transfection, cells were seeded one day prior to transfection. Then, 3 μL of Lipofectamine2000 was diluted in 100 μL of Opti-MEM, and 1 μg of DNA was diluted. Both solutions were gently mixed via vortexing and combined. The final transfection solution was mixed gently and incubated for 5 min at RT to allow Lipofectamine2000:DNA complex formation before transfection. For transfection with PEI, 293T cells were seeded one day before. Next, 10 μL of 10 mM PEI (pH 6.8) was diluted in 60 μL of DPBS, and 4 μg of DNA was diluted. Both solutions were mixed gently through vortexing and combined before incubation for 5 min at RT to allow PEI:DNA complex formation before transfection.

### 2.4. RNA Interference

Cells were transfected with the respective siRNAs using HiPerFect transfection reagent (Qiagen, Hilden, Germany) according to the manufacturer’s instructions for the fast-forward transfection of adherent cells. The siRNAs were as follows: non-targeting control siCtl with no mammalian homology (Luciferase, CDS), 5′-UUCUCCGAACGUGUCACGUdTdT-3′ (Microsynth AG, Balgach, Switzerland) [21]; human Aurora B kinase-specific siRNA (CDS), 5′-GGUGAUGGAGAAUAGCAGUdTdT-3′ (Microsynth AG) [22]; human Survivin-specific siRNAs–siSurv/siSurv1 (3′-UTR), 5′-AAGAAGAGCACAGUUGAAACAUCA-3′ (B02, Custom Stealth siRNA, Invitrogen/Thermo Fisher Scientific, Dreieich, Germany); siSurv2 (5′-UTR), 5′-GAAUGUGUCUGGACCUCAUGUUGUU-3′ (F02, Invitrogen/Thermo Fisher Scientific, Dreieich, Germany); and siSurv3 (CDS), 5′-GCAUUCGUCCGGUUGCGCUTT-3′ (BIRC5-5, Qiagen, Hilden, Germany). Cells were seeded in a well of a 6-well plate with 2 mL of medium for 24 h before transfection. An appropriate amount of the siRNA stock solution was diluted in 100 μL of Opti-MEM (Invitrogen/Thermo Fisher Scientific, Dreieich, Germany; usually 2 μL of a 20 μM stock solution, resulting in a final concentration of 20 nM in the medium). Ten μL of HiPerFect transfection reagent was added to the diluted siRNA, and the solution was mixed through vortexing. After incubation for 5 min at RT to allow the formation of transfection complexes, the transfection mix was added drop-wise to the cells, and the plate was swirled gently. The cells were incubated with the transfection complexes under normal growth conditions. If applicable, the cells were irradiated as indicated after 48 h and analyzed after another 24 h.

### 2.5. Irradiation and Inhibitor Treatment

Cells were exposed to different X-ray irradiation doses delivered via a CIX2 cabinet (Philips Constant Potential X-ray System MG160, Hamburg, Germany) and returned to the humidified incubator for the indicated duration. The PIP box inhibitor T2AA (Sigma-Aldrich/Merck KGaA, Darmstadt, Germany) was used at a final concentration of 40 μM. Camptothecin (CPT) was used at a final concentration of 1 μM. Hydroxyurea (HU) was used in a final concentration of 0.5 mM. Colcemid was used at a final concentration of 0.08 μg/mL to arrest the cell cycle during metaphase. Inhibitors as well as standard chemicals were purchased from Sigma-Aldrich/Merck KGaA, Darmstadt, Germany unless stated otherwise.

### 2.6. Immunofluorescence

Cells were seeded either in 8-well-μ-97 slide-ibi Treat (ibidi^®^, Gräfelfing, Germany) or in 3 cm microscopic glass bottom dishes (MatTek Corporation, Ashland, MA, USA) and transfected with calcium phosphate after 24 h. For immunofluorescence (IF) staining, cells were fixed with Roti^®^-Histofix 4% (Carl Roth, Karlsruhe, Germany) for 20 min at RT, permeabilized, and blocked with 5% normal goat serum (NGS) in 0.3% (*w*/*v*) Triton X-100/PBS for 30 min at RT. Cells were incubated with primary antibodies diluted in 1% BSA/0.3% Triton X-100/DPBS overnight at 4 °C. The antibodies used were as follows: human CREST serum (Antibodies Incorporated, Davis, CA, USA, 15-234; 1:400), rabbit anti-53BP1 (Novus Biologicals, Centennial, CO, USA, NB100-304; 1:2000), rabbit anti-Aurora B (Sigma Aldrich/Merck KGaA, Darmstadt, Germany, A5102; 1:2000), mouse anti-pATM (Santa Cruz Biotechnology, Heidelberg, Germany, sc-47739; 1:500), mouse anti-Borealin (MBL/Biozol, Eching, Germany, M147-3; 1:200), rabbit anti-CENP-C (Biozol, Eching, Germany, A06766-1; 1:100), rabbit anti-CENP-F (Novus Biologicals, Centennial, CO, USA, NB500-101; 1:750), mouse anti-cyclin A2 (Santa Cruz Biotechnology, Heidelberg, Germany, sc-271682; 1:100), rabbit anti-pDNA-PKcs (Abcam, Cambridge, UK, ab18192; 1:1000), mouse anti-DSN1 (Novus Biologicals, Centennial, CO, USA, 2A7; 1:800), mouse anti-HA (BioLegend, San Diego, CA, USA, 901501; 1:1000), mouse anti-Hec1 (GeneTex, Irvine, CA, USA, GTX70268; 1:1000), rabbit anti-INCENP (NEB/Cell Signaling, New England BioLabs, Frankfurt am Main, Germany, 2807; 1:400), mouse anti-myc (NEB/Cell Signaling, New England BioLabs, Frankfurt am Main, Germany, 2276; 1:1500), mouse anti-NSL1 (Origene, OTI4D3; 1:800), mouse anti-PCNA (NEB/Cell Signaling, New England BioLabs, Frankfurt am Main, Germany, 2586; 1:3200), rabbit anti-Survivin (Novus Biologicals, Centennial, CO, USA, NB200-201; 1:300), and mouse anti-γH2AX (BioLegend, San Diego, CA, USA, 613402; 1:10,000). Cells were washed three times with DPBS and incubated with appropriate secondary antibodies conjugated with a fluorescent dye (goat anti-mouse or goat anti-rabbit conjugated with Alexa Fluor dyes AF488 (green), AF568 (red), and AF633 (deep red, Invitrogen/Thermo Fisher Scientific, Dreieich, Germany) diluted in 1% BSA/0.3%Triton X-100/DPBS for 1 h at RT. For additional staining, cells were incubated with HCS CellMask™ Deep Red Stain (Thermo Fisher Scientific, Dreieich, Germany, H32721, 1:5000) and Hoechst 33342 (10 mg/mL in H_2_O, Applichem, Darmstadt, Germany, A0741, 1:1000) in DPBS (1% BSA, 0.3% Triton) for 1 h at RT.

### 2.7. Subcellular Fractionation

The Subcellular Protein Fractionation Kit (Thermo Fisher Scientific, Dreieich, Germany) was used to obtain cytoplasmic, membrane, nuclear soluble, chromatin-bound, and cytoskeletal protein extracts, according to the manufacturer’s instructions. Chromatin extraction was performed using a chromatin extraction kit (Abcam, Cambridge, UK) according to the manufacturer’s instructions.

### 2.8. Immunoprecipitation

Cells were seeded in 10 cm dishes, and co-transfection was performed 24 h after seeding. Whole-cell extracts were prepared 24 h after transfection by scraping the cells from the dish, washing the cell pellet in ice-cold PBS, and extraction with a 1 mL interaction buffer (50 mM of Tris at pH 8, 150 mM of NaCl, 5 mM of EDTA, 0.5% NP40, 1 mM of DTT, 1 mM of PMSF, and 1× complete protease inhibitor cocktail, Roche, Basel, Switzerland), followed by sonication (15 s at a 95% amplitude) using a Sonopuls mini20 device (Bandelin, Berlin, Germany). The lysates were centrifuged (15,000× *g*, 15 min, 4 °C), and the supernatant was transferred to a new reaction tube. The input samples from the lysates were stored separately. Immunoprecipitation (IP) was performed using the μMACS isolation kits for GFP- or FLAG-tagged proteins from Miltenyi Biotec (Bergisch Gladbach, Germany). The eluates and input samples were subjected to sodium dodecyl sulfate–polyacrylamide gel electrophoresis (SDS-PAGE) and immunoblotting. For chromatin–immunoprecipitation (ChIP), chromatin extraction was performed prior to immunoprecipitation, as described above, and one-third of the chromatin extract was used as an input control. The commercial antibodies used were rabbit anti-GFP (Santa Cruz Biotechnology, Heidelberg, Germany, sc-8334; 1:2000) and mouse anti-FLAG (Sigma-Aldrich/Merck KGaA, Darmstadt, Germany, F3165; 1:2000).

### 2.9. SDS-PAGE and Immunoblotting

Protein concentrations were determined using a Bio-Rad Protein Assay System (Bio-Rad Laboratories, Feldkirchen, Germany). Equal amounts of protein were resolved via SDS-PAGE using a 10% acrylamide gel and subsequently transferred onto either a polyvinylidene difluoride (PVDF) membrane (Amersham Hybond, GE Healthcare, München, Germany) or a nitrocellulose membrane (Amersham Protran; GE Healthcare München, Germany) using a PerfectBlue™ tank electro blotter (Peqlab, Erlangen, Germany) at 350 mA for 90 min. To minimize non-specific binding, the membranes were blocked with 5% (*w*/*v*) non-fat dried milk powder in TBST for 30 min at RT. The membranes were probed with primary antibodies diluted in a blocking solution overnight at 4 °C and washed three times with TBST before they were incubated with HRP-conjugated secondary antibodies (1:10,000, NXA931 and NA934, GE Healthcare, München, Germany) diluted in a blocking solution for 1 h at RT. The bound antibodies were visualized using enhanced chemiluminescence (ECL) with ECLplus Western Blotting Substrate from Pierce (Thermo Fisher Scientific, Dreieich, Germany), according to the manufacturer’s instructions. After incubation with the substrate, the signals were detected using a ChemiDoc MP Imaging System (Bio-Rad Laboratories, Feldkirchen, Germany). The commercial antibodies used were as follows: rabbit anti-Aurora B (Sigma-Aldrich/Merck KGaA, Darmstadt, Germany, A5102; 1:2000), mouse anti-Borealin (MBL/Biozol, Eching, Germany, M147-3; 1:500), rabbit anti-53BP1 (Novus Biologicals, Centennial, CO, USA, NB100-304; 1:1000), mouse anti-Flag (Sigma-Aldrich/Merck KGaA, Darmstadt, Germany, F3165; 1:2000), mouse anti-GAPDH (Santa Cruz Biotechnology, Heidelberg, Germany, sc-47724; 1:1000), rabbit anti-GFP (Santa Cruz Biotechnology, Heidelberg, Germany, sc-8334; 1:2000), mouse anti-Histone H3 (Abcam, Cambridge, UK, ab195277; 1:1000), rabbit anti-INCENP (NEB/Cell Signaling, New England BioLabs, Frankfurt am Main, Germany, 2807; 1:1000), rabbit anti-Lamin A/C (NEB/Cell Signaling, New England BioLabs, Frankfurt am Main, Germany, 2032, 1:1000), mouse anti-myc (NEB/Cell Signaling, New England BioLabs, Frankfurt am Main, Germany, 2276; 1:1000), mouse anti-PCNA (NEB/Cell Signaling, New England BioLabs, Frankfurt am Main, Germany, 2586; 1:100), rabbit anti-Pol η (Novus Biologicals, Centennial, CO, USA, NB100-60424; 1:1000), rabbit anti-Survivin (Novus Biologicals, Centennial, CO, USA, NB200-201; 1:1000), mouse anti-Tubulin (Sigma-Aldrich/Merck KGaA, Darmstadt, Germany, T6074; 1:8000), and mouse anti-γH2AX (BioLegend, San Diego, CA, USA, 613402; 1:5000).

### 2.10. DNA Fiber Assay

Exponentially growing cells, seeded in a 6-well plate, were initially pulse-labeled with the first thymidine analog. Chlorodeoxyuridine (CldU, Sigma-Aldrich/Merck KGaA, Darmstadt, Germany) was added to the cell culture at a final concentration of 25 μM, and the cells were incubated for 20 min under normal growth conditions. Thereafter, iododeoxyuridine (IdU, Sigma-Aldrich/Merck KGaA, Darmstadt, Germany) was added in excess at a final concentration of 250 μM, and the cells were again incubated for 20 min under normal growth conditions. After double labeling, the cells were trypsinized, pelleted at 300× *g* for 5 min at 4 °C, and washed twice with ice-cold DPBS. The cell pellet was resuspended in a small volume of cold DPBS (0.25–0.5 mL), and the cells were counted and diluted to a final concentration of 5 × 10^5^ cells/mL in cold DPBS and kept on ice. Then, 2 μL of the cell suspension was spotted on the top of uncoated microscope slides and air-dried for 5–7 min until the drop was sticky but not completely dry. Subsequently, 7 μL of spreading buffer (mM Tris-HCl at pH 7.4, 50 mM EDTA, 0.5% (*w*/*v*) SDS) was applied on top of the cell suspension and then mixed through gentle stirring with a pipette tip and incubated for 2 min. Following cell lysis, the slides were tilted slightly (approximately to 15°), and the drops were allowed to run down the length of the glass slide and spread the DNA fibers along the slide. Once dried, the DNA spreads were fixed by incubating the slides for 10 min in a 3:1 solution of methanol/acetic acid in a glass staining jar. The slides were dried and stored at 4 °C. For the immunostaining of the incorporated halogenated thymidine analogs, the slides were washed twice with H_2_O for 5 min in a glass staining jar. dsDNA was denatured by covering the glass slides with 2.5 M HCl for 75 min in a staining tray. Afterwards, the slides were rinsed twice with DPBS, washed twice with blocking solution (DPBS, 1% BSA, 0.1% Tween 20) for 5–10 min each, and then incubated in blocking solution for 30–60 min. Excess blocking solution was removed using a paper towel, and the slides were placed horizontally in a humidified chamber. Subsequently, 115 μL of an antibody mix of rat anti-BrDU (CldU detection; dilution: 1:1000; Abcam, Cambridge, UK, ab3626) and mouse anti-BrdU (IdU detection; dilution: 1:500; BD Bioscience, Heidelberg, Germany Clone B44, 347580) in blocking solution were added and incubated under a coverslip for 1 h at RT. After the coverslips were removed by gently moving down the slide without applying force, the slides were rinsed 3× with DPBS and fixed for 10 min in 4% paraformaldehyde (PFA). The slides were rinsed again 3× with DPBS and then washed 3× with blocking solution for 1, 5, and 25 min. Then, 115 μL of a mix of secondary antibodies (anti-rat coupled with AF568 and anti-mouse with AF488) in blocking solution were added and incubated for 1.5–2 h while protected from light. After removing the coverslips, the slides were rinsed twice with DPBS, washed 3× with blocking solution for 1, 5, and 25 min, and again rinsed twice with DPBS. Finally, they were mounted using FluorSave™ reagent (Calbiochem/Merck KGaA, Darmstadt, Germany) and stored at −20 °C for subsequent confocal microscopy. The lengths of the CldU and IdU tracks were measured using ImageJ 1.47v, and µm-values were converted into kb values to calculate the replication fork speed in kb/min.

### 2.11. Proximity Ligation Assay

Proximity ligation assay (PLA) staining was performed with Duolink^®^ In Situ PLA^®^ Probes mouse/rabbit together with the Duolink^®^ In Situ Detection Reagents Orange (Sigma-Aldrich/Merck KGaA, Darmstadt, Germany), following the manufacturer’s instructions [16]. The antibodies used were rabbit anti-Aurora B (Sigma-Aldrich/Merck KGaA, Darmstadt, Germany, A5102; 1:2000), mouse anti-Borealin (MBL/Biozol, Eching, Germany, M147-3; 1:500), rabbit anti-INCENP (NEB/Cell Signaling, 2807; 1:1000), mouse anti-DNA Ligase I (Merck, clone 5H5/MABE1905; 1:1000), mouse anti-PCNA (NEB/Cell Signaling, 2586; 1:1000), rabbit anti-PCNA (Santa Cruz Biotechnology, P10, sc-56; 1:500), and rabbit anti-Survivin (Novus Biologicals, Centennial, CO, USA, NB200-201; 1:1000).

### 2.12. EdU Incorporation and Staining

The Click-iT™ EdU Alexa Fluor™ Imaging Kit (Thermo Fisher Scientific, Dreieich, Germany) was used to identify cells that were actively involved in DNA replication by incorporating the thymidine analog ethynyl deoxyuridine (EdU) into newly synthesized DNA and labeling it with an Alexa Fluor fluorescent dye via click chemistry, according to the manufacturer’s instructions.

### 2.13. Metaphase Chromosome Spreading

Metaphase chromosome spreading was used to visualize gaps and breaks in metaphase chromosomes following replication stress. The cells were cultivated and treated accordingly. Afterwards, the cells were incubated for 2 h with 0.08 μg/mL of colcemid (Sigma-Aldrich/Merck KGaA, Darmstadt, Germany) to arrest them in metaphase and detached through washing with DPBS. The collected cells were centrifuged for 7 min at 130× *g* and then incubated with 5 mL of preheated (37 °C) sodium citrate for 10 min. Afterwards, the cells were centrifuged, and the pellet was fixed in total three times with 5 mL of drop-wise fixation solution (150 mL of methanol + 50 mL of acetic acid) while vortexing. The volume of the fixation solution was reduced by gently removing the supernatant to obtain a highly concentrated solution. Spreading was performed by dropping the cell solution onto a microscope slide at a distance of approximately 50 cm. The slides were then dried overnight at RT and stained with Giemsa staining solution (10 mL of Giemsa and 40 mL of phosphate buffer).

### 2.14. Aurora B Kinase Assay

The analysis of Aurora B kinase activity to verify new substrates was performed using the Aurora B kinase assay (Promega, Walldorf, Germany) comprising Aurora B kinase, native swine myelin basic protein (MBP) as a substrate, and the ADP-Glow^TM^ Kinase Assay to quantify ADP formed from a kinase reaction (Figure S12A). After the enzymatic reaction was completed, the remaining ATP was depleted before ADP was converted to ATP, which was then turned into light via luciferase. Here, the ADP-Glow^TM^ assay was modified to be performed in 96-well plates instead of 384-well plates. Therefore, the kinase reaction volume was increased to 25 µL and incubated at RT for 60 min before the addition of 25 µL of ADP-Glow^TM^ reagent. The reaction mix was incubated for 40 min at RT, followed by the addition of 50 µL of kinase detection reagent and successive incubation for 30 min at RT before a luminescent readout using a plate reader (Glomax Multi, Promega) with an integration time of 0.5 s. A calibration curve was established by measuring 11 solutions containing different ADP and ATP ratios from 100% ADP to 100% ATP in steps of 10% at a volume of 25 µL and 25 µM conc (Figure S12B). The solutions were incubated for 60 min at RT before the ADP-Glow^TM^ assay was performed as described above. To validate POLDIP2 as a substrate of Aurora B kinase, reaction solutions containing ATP (25 µM), recombinant human POLDIP2/PDIP38 (Tag: DKK/myc, source: 293T cells, Origene, TP300053; 2.5 µM), and increasing amounts of Aurora B kinase (0.1 ng to 200 ng) were incubated for 60 min at RT before the assay was performed. The same experiment was performed using MBP (2.5 µM) as the positive control. The experiments were performed in triplicate.

### 2.15. Software and Statistical Analyses

Images of the Western blots and Coomassie gels were acquired using the ChemiDoc Imaging System v2.2.0.08 (Bio-Rad Laboratories) and quantified via densitometric analysis using Fiji [23]. The molecular modeling of POLDIP2 was performed using AlphaFold AF2, https://alphafold.ebi.ac.uk/ (accessed on 20 September 2022). For experiments with *p*-values, unpaired *t*-tests were performed. The *p*-values represent data obtained from independently repeated experiments (number of replicates, number of measurements, and total cell number, as indicated). *p*-values <0.05 were considered significant (* *p* < 0.05; ** *p* < 0.001; *** *p* < 0.0001). All analyses and graphs were generated using Graph Pad Prism, version 9. Figures and schemes were created using BioRender.com.

## 3. Results

### 3.1. Irradiation Induces Survivin Foci Formation in Interphase Nuclei

**Figure 1:**
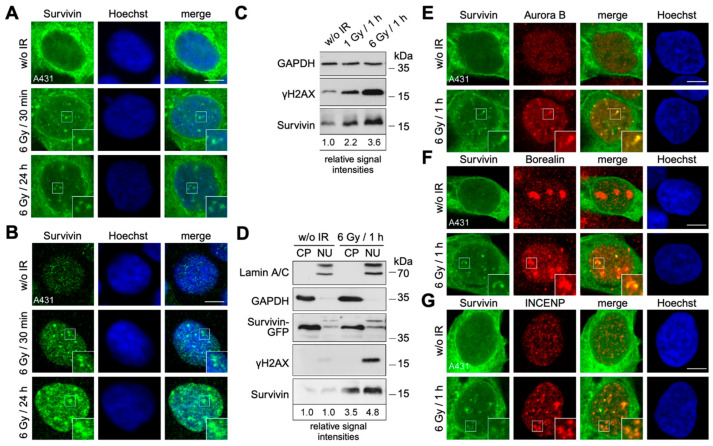
Irradiation increases Survivin expression and relocation to nuclear foci as part of the CPC. (**A**,**B**) A431 cells stably expressing Survivin-GFP (**A**), as well as parental A431 cells (**B**), were irradiated with 6 Gy, fixed 30 min, and 24 h after IR, permeabilized and subjected to confocal microscopy. Cells in (**B**) were additionally immunostained with a Survivin-specific antibody (green). DNA was stained with Hoechst (blue). Non-irradiated cells (w/o IR) were used as controls. Scale bar: 7.5 μm. (**C**) A431 cells stably expressing Survivin-GFP were irradiated with 1 or 6 Gy and lysed 1 h after IR for immunoblot analysis. (**D**) A431 cells stably expressing Survivin-GFP were irradiated with 6 Gy and, 1 h after IR, subjected to subcellular fractionation, separating cytoplasm (CP) from nuclei (NU), and subsequent immunoblot analysis. GAPDH served as loading control for the CP fraction and Lamin A/C for the NU fraction, respectively. The relative signal intensities of Survivin bands were quantified via a densitometric analysis using ImageJ and normalized to GAPDH (**C**) or GAPDH and Lamin A/C (**D**). The depicted values represent the relative increase in Survivin levels after IR. (**E**–**G**) A431 cells stably expressing Survivin-GFP were irradiated with 6 Gy, fixed 1 h after IR, and permeabilized. Cells were immunostained with antibodies specific to Aurora B (**E**), Borealin (**F**), or INCENP (**G**), respectively (red), and subjected to confocal microscopy. DNA was stained with Hoechst (blue). Non-irradiated cells (w/o IR) were used as controls. Scale bar: 7.5 μm. The insets show higher magnifications of the areas outlined in the main panels. Representative images are shown.

The irradiation (IR) of the different epithelial cancer cell lines A431 and HeLa, as well as the osteosarcoma cell line U2OS, resulted in rapid (30 min) and persistent (24 h) Survivin accumulation in the nuclear foci of interphase cells (Figure 1A,B and Figure S1). Notably, in non-irradiated cells, Survivin-GFP was mainly localized in the cytoplasm and rapidly translocated into the nucleus following IR (Figure 1A). However, some residual, rather small nuclear Survivin foci can also be observed in non-irradiated cells. Similar to its physiological CPC-binding partners Aurora B, INCENP, and Borealin, endogenous Survivin preferentially resides in the nucleus, and Survivin nuclear foci form and intensify after IR (Figure 1B). Also here, some endogenous Survivin foci can also be observed in non-irradiated cells, although they are hard to distinguish from the staining background. But also here, it is quite evident that the number of nuclear Survivin foci decreased over time, whereas their size increased. Immunoblot analysis verified a dose-dependent increase in endogenous expression, like the DNA damage marker γH2AX (Figure 1C). Fractionation revealed an induction of Survivin in both cytoplasm and, more prominently, the nucleus (Figure 1D). Notably, although stably expressed Survivin-GFP remained unaffected due to its constitutive promoter, its intracellular translocation was clearly reflected by a reduction in the cytoplasm and a corresponding increase in the nucleus.

A detailed analysis of the foci kinetics revealed that low-dose IR (0.5 Gy) immediately initiated the relocation of Survivin to the nucleus and the formation of several distinct foci (Figure S2A,B). The latter decreased in number and intensity over time, whereas those induced via moderate or high doses became more pronounced (Figure S2). Endogenous immunoblot analysis revealed an increase as soon as 15 min after IR, which intensified and persisted for up to 24 h (Figure S2C). Notably, γH2AX induction was observed as soon as 5 min after IR. γH2AX foci formation followed comparable kinetics dependent on the radiation dose. However, it was obvious that the γH2AX foci clearly differed from the Survivin nuclear foci that, rather, localized in close proximity to each other (Figure S2A,B). Notably, as γH2AX spreads up to 1–2 Mbp from the DNA break, it is an indicator of DNA damage in general but not of the specific site of damage [7].

As previously mentioned, one of the most important roles of Survivin is the regulation of mitosis as part of the CPC [2]. This complex is composed of a scaffolding module consisting of Survivin, Borealin, and INCENP and an enzymatic component, Aurora B kinase. Most studies on the CPC and its functions have focused on mitotic cells, and only little is known about possible additional tasks during interphase. As Survivin formed nuclear foci in non-mitotic cells after IR, it was tempting to speculate as to whether other CPC members might be present at the same sites. The immunostaining of Aurora B kinase, Borealin, and INCENP indeed revealed an IR-induced accumulation in nuclear foci, perfectly co-localizing with Survivin-GFP (Figure 1E–G).

### 3.2. Survivin Accumulates in Centromeric Heterochromatin After Irradiation

**Figure 2:**
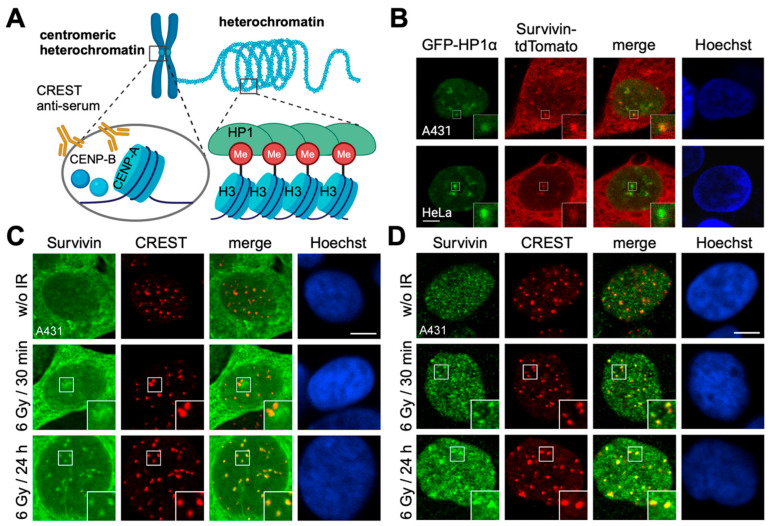
Survivin accumulates in centromeric regions after irradiation. (**A**) Schematic representation of centromeric heterochromatin. In contrast to transcriptionally silenced heterochromatin, mainly characterized by Heterochromatin protein-1 (HP1) bound to the methylated lysine 9 residue of histone H3 (H3K9me), constitutive or centromeric heterochromatin additionally relies on CENP-A as a stable centromere component and CENP-B that also belongs to the centromere and is also recognized by CREST. (**B**) A431 and HeLa cells were co- transfected with plasmids coding for EGPF-HP1α (green) and Survivin-tdTomato (red), permeabilized, fixed, and subjected to confocal microscopy. DNA was stained with Hoechst (blue). Scale bar: 7.5 μm. (**C**,**D**) A431 cells stably expressing Survivin-GFP (**C**), as well as parental A431 cells (**D**), were irradiated with 6 Gy, fixed 30 min and 24 h after IR, permeabilized, and immunostained with CREST serum, detecting centromeric chromatin (red) for confocal microscopy. Cells in (**D**) were additionally incubated with a Survivin-specific antibody (green). DNA was stained with Hoechst (blue). Non-irradiated cells (w/o IR) were used as controls. Scale bar: 7.5 μm. The insets show higher magnifications of the areas outlined in the main panels. Representative images are shown.

The CPC was originally defined by the characteristic localization pattern its members exhibit during mitosis [2]. It localizes to and is the key regulator of inner centromere organization and function during mitosis, and as such, it is associated with a very unique chromatin structure. Consequently, we aimed to assign the localization of IR-induced foci to specific chromatin architectures. Chromatin can be differentiated into loosely packed euchromatin containing actively transcribed genes and tightly packed regions termed heterochromatin homing inactive genes and repetitive DNA sequences [24,25]. As a key component of heterochromatin, Heterochromatin protein-1 (HP1) directly binds to the methylated lysine 9 residue of histone H3 (H3K9me), which is a hallmark histone modification of transcriptionally silenced chromatin (Figure 2A) [25]. Interestingly, HP1 also links centromeric heterochromatin to centromere cohesion, and the mitosis-specific phosphorylation of HP1α regulates its ability to bind chromatin. Indeed, by utilizing eGFP-tagged HP1α, we could allocate nuclear Survivin-tdTomato foci to heterochromatin regions (Figure 2B), and co-localization increased with the radiation dose (Figure S3A). More specifically, IR-induced Survivin foci clearly co-localized with centromeric heterochromatin labelled with CREST serum in different cell lines, most prominently after high-dose IR (Figure 2C,D and Figure S3B) CREST autoimmune sera are derived from scleroderma patients and directed against human centromere proteins, mainly CENP-A, -B, and -C in varying stoichiometries [26]. In particular, CENP-A is a centromere-specific histone H3 variant characteristic of centromeric heterochromatin (Figure 2A and Figure S4A) [27]. Whereas CENP-A is a stable centromere component, CENP-B and CENP-C, inter alia, exhibit distinct cell cycle-specific centromere-binding [28]. CENP-B also belongs to the centromere, whereas CENP-C and most other CENPs (CENP-H to -X) are part of the inner kinetochore module—the constitutive centromere-associated network (CCAN) complex, which is also known to interact with the neighboring CPC (Figure S4A). In contrast, CENP-E and CENP-F were localized in the outer kinetochore. Indeed, Survivin co-localized with CENP-C at the inner kinetochore (Figure S4B) but not with the essential kinetochore-associated NDC80 (Nuclear division cycle 80) complex components CENP-F (Figure S4C) and Hec1 (highly expressed in cancer protein-1; Figure S4D), or the MIS12 outer kinetochore complex components DSN1 (Figure S4E) and NSL1 (Figure S4F).

### 3.3. Survivin Depletion Results in Increased H2AX Phosphorylation

**Figure 3:**
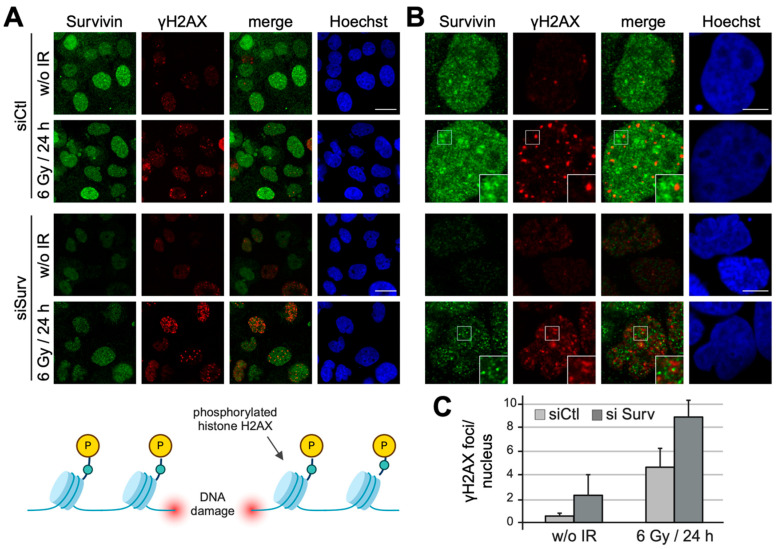
H2AX phosphorylation increases after Survivin depletion. (**A**,**B**) A431 cells were transfected with 20 nM of non-targeting control siRNA (siCtl) and Survivin-specific siRNA (siSurv), irradiated with 6 Gy 48 h after transfection, and fixed 24 h after IR. As indicated in the scheme, the phosphorylation of histone H2AX occurs in the vicinity of damaged DNA. Cells were immunostained with antibodies specific to phosphorylated H2AX (γH2AX, pS139) (red) and Survivin (green) and analyzed via confocal microscopy in two different magnifications. DNA was stained with Hoechst (blue). Non-irradiated cells (w/o IR) were used as a control. The insets show higher magnifications of the areas outlined in the main panels. Representative images are shown. Scale bars: 25 μm (**A**) and 7.5 μm (**B**). (**C**) For quantification, images were processed with ImageJ. Each bar represents the mean ± SD of residual γH2AX foci per nucleus of two repeated experiments (200–300 evaluated nuclei each) of cells treated with non-targeting control siRNA (siCtl) or Survivin-specific siRNA (siSurv).

As already mentioned, Survivin’s well-known roles in apoptosis inhibition and mitotic progression both indirectly foster a radio-resistant phenotype. Recent studies, however, suggest an additional, more direct involvement in the DDR, inter alia, by binding and modulating the DNA-dependent protein kinase catalytic subunit (DNA-PKcs) [29]. Consequently, the DDR capacity was analyzed in cells after Survivin depletion by quantifying γH2AX foci 24 h after IR. Notably, in Survivin-depleted cells, residual endogenous Survivin expression was slightly enhanced after IR compared to non-irradiated depleted cells (Figure 3A,B). Indeed, the number of radiation-induced γH2AX foci was considerably increased in Survivin-depleted cells (Figure 3A–C). Quantification revealed an almost doubling of the foci number after Survivin knockdown, as well as a slightly increased number of γH2AX foci in non-irradiated cells (Figure 3C). Interestingly, although they resemble each other in size, intranuclear distribution and inducibility following IR, γH2AX, and Survivin foci clearly showed no co-localization. Similarly, Survivin nuclear foci did not overlap with p53 binding protein 1 (53BP1) foci (Figure S5B), which are known to reside in close proximity to γH2AX (Figure S5A), or with phosphorylated ataxia telangiectasia mutation (pATM) directly associated with the site of DNA damage (Figure S5A,C,D).

Immunoblot analyses of Survivin- and Aurora B-depleted and irradiated cells revealed a two-fold increase in phosphorylated H2AX 4 h after IR in the lysates of cells (Figure S2D,E). However, in lysates of Survivin-knockdown cells, a prominent increase in γH2AX was detected even without IR, and IR with 6 Gy did not further enhance H2AX phosphorylation. Notably, Aurora B depletion was not as effective as the Survivin depletion. Increased levels of H2AX phosphorylation were detected after IR and appeared to be slightly enhanced in lysates depleted of Aurora B kinase (Figure S2E). Notably, compared to a complete Survivin knockdown (Figure S2D), residual endogenous Survivin counteracted H2AX phosphorylation in non-irradiated cells (Figure S2E). A ubiquitinated form of γH2AX at 25 kDa was detected most prominently in lysates of irradiated control cells that were treated, and it disappeared following knockdown (Figure S2D,E).

### 3.4. The CPC Co-Localizes to Replication Sites and Affects the Replication Machinery

**Figure 4:**
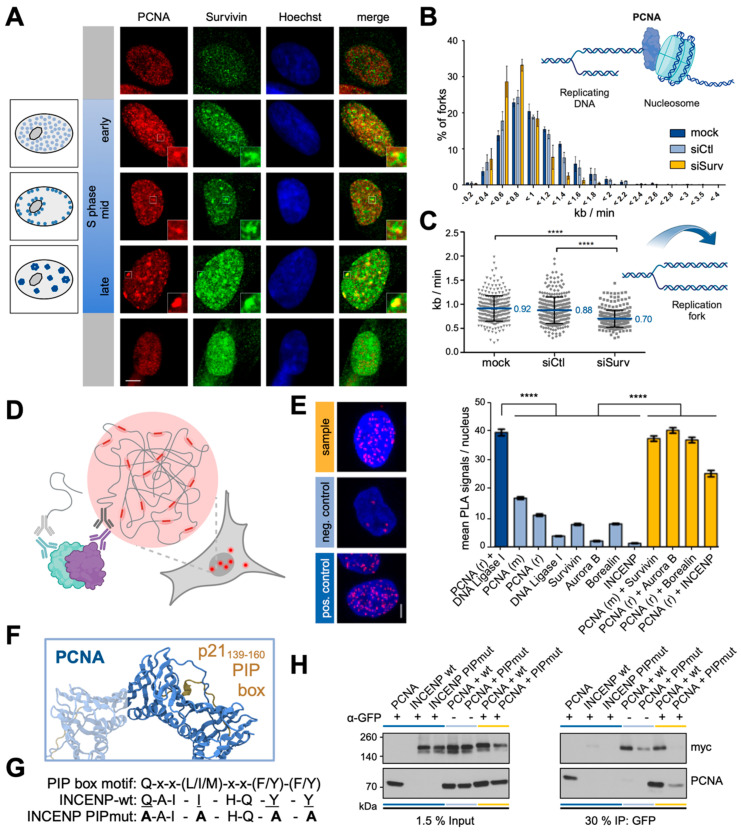
Interaction of PCNA with the CPC enables unhindered replication fork progression. (**A**) WI-38 cells were fixed, permeabilized, and immunostained for Survivin (green) and PCNA (red). DNA was stained with Hoechst (blue). Scale bar: 5 µm. Insets, higher magnifications of the areas outlined in the main panels. Left: schematic representation of characteristic patterns of PCNA distribution in S-phase substages. (**B**,**C**) Survivin knockdown reduces replication fork speed. A431 cells were transfected with siRNA specific to Survivin (siSurv), non-targeting siRNA (siCtrl), or without siRNA (mock) as a control. Then, 72 h after transfection, cells were sequentially pulse-labeled with CldU and IdU for 20 min each. Cells were harvested, and the DNA fiber assay was performed. The length of stained fibers from elongating forks was measured. Results are depicted as a frequency distribution. Means ± SD from three independent experiments with 300 fibers analyzed per experiment are shown. (**C**) Dot plot of the mean replication fork speed of one representative data set out of three independent experiments, as shown in (**B**). Bars: means ± SD (*n* = 300 fibers of each group). ****: *p* < 0.0001. (**D**,**E**) CPC proteins interact with PCNA as analyzed via a proximity ligation assay (PLA, (**D**)) in HeLa cells (**E**). Representative images are shown. Each red spot represents a single PLA interaction signal. DNA was stained with Hoechst (blue). Scale bar: 5 µm. The number of PLA signals per nucleus from >150 cells was quantified with ImageJ. Bars: means ± SEM of a positive control (dark blue), negative controls (light blue), and interaction samples (yellow). ****: *p* < 0.0001. (**F**–**H**). INCENP binds to PCNA via a PIP-box motif. Exemplary cartoon presentation of a PCNA monomer (blue) as part of the homotrimer (transparent) bound to the p21 PIP-box peptide (brown, 1AXC) (**F**). Overview of the PIP-box consensus, the same motif in wildtype INCENP (wt) and the mutated (PIPmut) PIP-box motif (mutated aa, highlighted in bold) (**G**). 293T cells were transfected (or co-transfected) with GFP-PCNA, myc-INCENPwt, or myc-INCENP PIPmut, as indicated (**H**). Single transfections are highlighted in dark blue. Lysates were immunoprecipitated (IP) with magnetic α-GFP beads (yellow) or uncoupled beads (light blue) and immunoblotted with indicated antibodies. Representative images are shown.

Although our data suggested a functional implication in the DDR, Survivin nuclear foci were not associated with bona fide DNA repair factors (Figure S5) but, rather, concentrated in heterochromatic regions characterized by the presence of HP1 (Figure 2 and Figure S3). HP1 is well known for its role in different cellular processes, including transcriptional and cell-cycle regulation, centromere/telomere maintenance, splicing, DDR, and replication [30]. Indeed, DNA replication and repair are closely linked processes that preserve genomic stability and ensure the faithful transmission of genetic information. DNA repair mechanisms play a crucial role in preventing replication errors and alleviating replication stress. Additionally, DNA damage is repaired during replication, facilitating replication fork protection and restart. Alongside the spindle checkpoint in mitosis, the two primary cell-cycle checkpoints that coordinate the cell’s response to DNA damage occur toward the end of G1 and at the G2/M transition in interphase. Notably, some residual (endogenous) Survivin foci could also be detected in non-irradiated interphase nuclei (Figure 1, Figure 2 and Figure 3, Figures S1–S3 and S5). Consequently, the interphase localization of Survivin was analyzed in exponentially growing human normal embryonic WI-38 fibroblasts, where replication is not hampered due to chromosomal aberrations as in cancerous cell lines (Figure 4A). In brief terms, processive chromosomal replication requires ring-shaped sliding clamp factors that encircle the DNA to anchor polymerases and replisome proteins. PCNA, the homo-trimeric eukaryotic sliding clamp (Figure S6A,F), reveals a characteristic temporal distribution pattern in S phase, where it marks distinct sites of DNA synthesis during replication (Figure 4A) [31]. In both G phases, PCNA was equally distributed over the whole nucleus and agglomerated to small, equally distributed foci in the early S phase. In the mid-S phase, these foci were located at the nuclear periphery, and in the late S phase, they were enlarged near the center of the nuclei. Finally, PCNA is displaced from the condensed chromosomes during mitosis. Interestingly, Survivin is already expressed in the early S phase and forms nuclear foci. While some of these Survivin foci were in close proximity or directly co-localized with PCNA, most of them did not seem to be related to PCNA. However, in the late S phase, the majority of Survivin foci co-localized with PCNA. In the subsequent G_2_ phase, Survivin expression and localization were similar to those observed in the late S phase. Moreover, in WI-38 normal fibroblasts, HeLa, and A431 cancer cells, Aurora B kinase was localized to the nucleus during the S phase and formed foci partially co-localized, or at least in very close proximity to PCNA (Figure S6C). As we already confirmed co-localization of Survivin with the other CPC proteins (Figure 1E–G), as well as with HP1 and CREST (Figure 2 and Figure S3), particularly after IR, we verified the intracellular patterns for a sub-selection of proteins, including HP1α and INCENP (Figure S6D), CREST and Aurora B (Figure S6E) or PCNA, HP1α and Aurora B (Figure S6F) in normal and cancer cell lines, respectively. As such, Survivin might exert its novel cellular function in DNA replication as part of the CPC, whose role is no longer restricted to mitosis. Consequently, we performed co-staining experiments with replication markers. A431 cells incorporated EdU for 20 min into nascent DNA, which was later visualized through EdU staining combined with CREST and Aurora B staining (Figure S7A). Replication sites were detected at the nuclear periphery, and more pronounced EdU foci were distributed throughout the nucleus, corresponding to cells in transition from the mid to late S phase (Figure 4A). Whereas the majority of Aurora B foci co-localized with CREST, they were also present at some replication sites, as well as in solitary foci lacking EdU or CREST. In particular, the latter indicates additional biological functions of this important kinase apart from replication, which we have not yet anticipated. Next, we utilized mouse NIH3T3 cells for immunostaining (Figure S7B), as these feature large pericentric heterochromatin clusters called Hoechst-dense chromocenters [24]. Here, replication sites could be visualized via PCNA staining, surrounded by CREST signals at the periphery. Likewise, Aurora B was concentrated at the chromocenters in close proximity to CREST and, in an even more pronounced manner, to PCNA.

To gain functional insights into this replicative Survivin function, we utilized the DNA fiber assay technique (Figure S8) to visualize fork velocities [32]. Here, the incorporation of halogenated thymidine analogs (chlorodeoxyuridine, CIdU, and iododeoxyuridine, IdU) allows the visualization of newly synthesized DNA in asynchronous cell populations at the level of single-spread DNA molecules (Figure S8A,C), as well as the analysis of replication fork speed and kinetics (Figure S8B). Microscopic images visualized a shorter tract length in Survivin-depleted cells than in control cells (Figure 4B,C). The quantification of the replication fork speed revealed a distribution between 0.2 and 4 kb/min, at a mean fork velocity of 0.6–1.2 kb/min (Figure 4B). The mean incorporation rate was significantly decreased in Survivin-depleted cells (0.70 ± 0.01 kb/min) compared to mock- (0.92 ± 0.02 kb/min) or siCtl-treated (0.88 ± 0.02 kb/min) cells (Figure 4C).

### 3.5. The CPC Co-Localizes with PCNA via INCENP’s PIP-Box Motif

To further validate that the CPC was indeed associated with the replisome via PCNA, an in situ proximity ligation assay (PLA) was performed to reveal endogenous protein–protein interactions in situ at distances of less than 40 nm (Figure S9 and Figure 4D). As DNA Ligase I and PCNA are established interaction partners [31], they served as positive controls (Figure 4E). Indeed, we revealed a close interaction between PCNA and all CPC members, as the numbers of discrete PLA signals per cell nucleus were significantly increased compared with the respective controls (Figure 4E). However, because of the relatively low spatial resolution of the PLA assay (40 nm), it is most likely that only one distinct CPC member directly interacts with PCNA while simultaneously being part of the CPC, which is, in its entirety, smaller than 40 nm, and hence out of resolution (Figure S6B). The distances between the CPC members were below 10 nm, whereas the PCNA trimer spans almost 90 nm in diameter (Figure S6A). Importantly, most interactions with PCNA are mediated via either an APIM (AlkB homolog 2 PCNA-interacting motif) or a PIP (PCNA-interacting protein) box motif in the partner protein (Figure S10A and Figure 4F) [31]. Indeed, a putative PIP-box motif was identified in INCENP via bioinformatic screening (^853^**Q**AI**I**HQ**YY**^860^, pivotal residues underlined; Figure S10B). This motif is located at its C-terminus, which is known to interact with Aurora B and is highly conserved (Figure S10C,D). Consequently, a myc-tagged INCENP-PIP box mutant (Figure 4G) was designed by exchanging the essential consensus residues with alanine (^853^QAIIHQYY^860 853^**A**AI**A**HQ**AA**^860^) and used for co-immunoprecipitation (Figure 4H). Indeed, the interaction with GFP-PCNA was severely hampered for myc-INCENP-PIPmut. Next, the inhibitor T2AA was used to interfere with the PCNA/PIP-box peptide interaction [33]. Here, PLA analysis was combined with EdU incorporation into newly synthesized DNA visualized using the Click-iTTM Kit to detect replicating cells (Figure S11). Briefly, HeLa cells were treated with 40 μM of the PIP-box inhibitor T2AA or DMSO as a control for 4 h. During the final 3 min of inhibitor treatment, 10 μM of EdU was added (Figure S11A). The PLA analysis was restricted to EdU-positive replicating cells (Figure S11B,C), and the mean signal intensity increased by 1.5-fold compared to that of the negative controls. In contrast, PLA signal intensities were significantly decreased following the T2AA treatment, indicating a hampered INCENP/PCNA interaction (Figure S11B,C). Notably, this effect correlated with lower EdU intensities (Figure S11B) resulting from less incorporation. These data now identify the PIP-box motif in INCENP as a functional binding site for PCNA and link the replication site recruitment of the CPC to INCENP.

### 3.6. Survivin Depletion Impedes the Cell’s Response to Replication Stress

**Figure 5:**
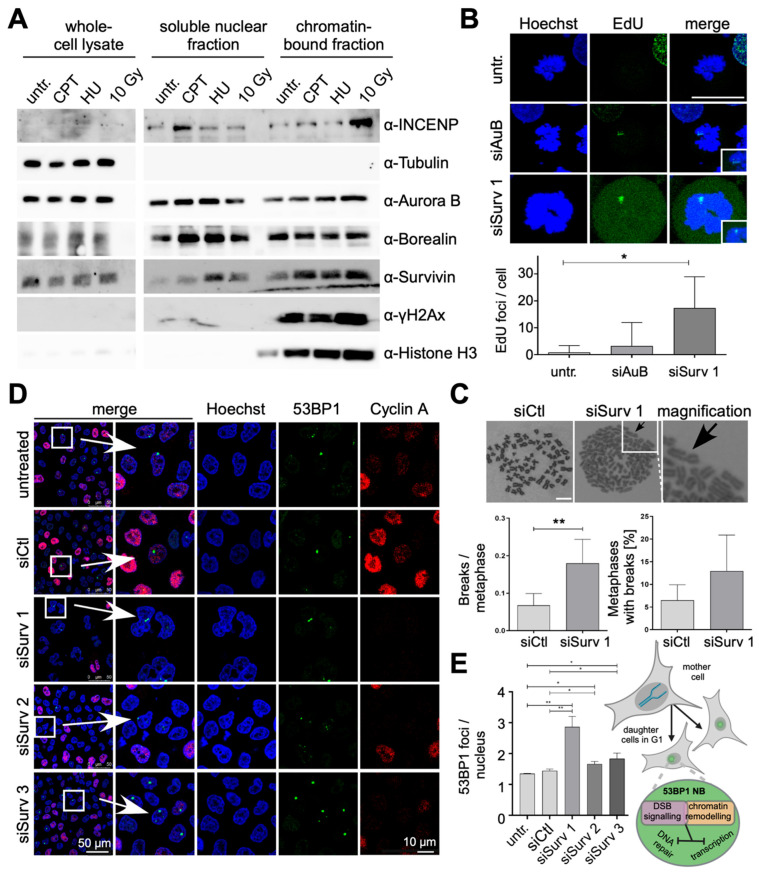
Survivin depletion impedes the cell’s response to replication stress. (**A**). Subcellular expression of the CPC after induction of replication stress; 293T cells were treated with different reagents inducing replication stress, including 1 µM of Camptothecin, 0.5 mM of Hydroxyurea, and IR with 10 Gy. Whole-cell extracts: and subcellular fractions were generated and subjected to immunoblot analysis with indicated antibodies. γH2AX served as a marker for DNA damage and repair, and Tubulin and Histone H3 served as loading controls for the cytoplasmic fraction and the chromatin fraction, respectively. (**B**). CPC depletion results in mitotic DNA synthesis. HeLa cells were transfected with siRNAi against Survivin or Aurora B, fixed, and permeabilized 48 h later. During the last 16 h of depletion, cells were incubated with RO-3306 to arrest them in G_2_ phase. Cells were stained with the Click-iT™ EdU Alexa Fluor™ 488 Imaging kit. DNA was stained with Hoechst (blue). Scale bar: 25 µm. Quantitative analysis of EdU foci in metaphase cells. Bars: mean number of EdU foci per cell ± SD in >50 cells. Data were analyzed via a *t*-test. *: *p* < 0.05. n = 3. (**C**). Survivin depletion leads to an increased number of breaks in metaphase chromosomes (black arrows). Brightfield images of metaphase spreads with a full set of chromosomes. Scale bar: 10 µm. Quantitative analysis of breaks/gaps per metaphase and of the number of metaphases with breaks. Bars: mean number of breaks per metaphase or the mean number of metaphases with breaks ± SD in >100 metaphases. Data were analyzed via a *t*-test. **: *p* < 0.01. n = 3. (**D**,**E**) Survivin depletion leads to an increase in 53BP1 nuclear bodies during the G_1_ phase. HeLa cells were transfected with Survivin-specific or non-targeting siRNA, fixed, and permeabilized after 48 h. Cells were immunostained with antibodies specific to 53BP1 (green) and cyclin A (red). DNA was stained with Hoechst (blue). Scale bars: 50 and 10 µm (**D**). Quantitative analysis of 53BP1 nuclear bodies in cells treated with Survivin-specific or non-targeting siRNA (**E**). Bars: mean number of 53BP1 nuclear bodies per nucleus ± SD in >100 cells. Data were analyzed via a *t*-test. **: *p* < 0.01; *: *p* < 0.05. n = 3.

The role of PCNA as a platform for recruiting DNA replication and repair factors has been well documented, in particular, to facilitate replication or manage replication stress [31]. In general, the latter is a condition in which stalled or slowly progressing replication forks interfere with timely and error-free S-phase completion, and it is a major source of structural chromosome instability. Consequently, to elucidate whether this also affects the members of the CPC, we first analyzed their expression and intracellular localization in response to different replicative stressors, and 293T cells were treated with either 1 μM of Camptothecin (CPT), 0.5 mM of Hydroxyurea (HU), or IR with 10 Gy. CPT is a topoisomerase inhibitor that frequently induces breaks at replication forks (direct stress inductor), and HU as an inhibitor of ribonucleotide reductase (RNR) causes dNTP depletion, replication fork arrest, and, ultimately, DNA breakage (indirect inductor). Ionizing radiation induces DSBs either directly or indirectly when single-strand DNA lesions block replicative polymerases, and it finally leads to replication fork collapse and, thus, to DSBs. All treatment modalities effectively induced DNA damage, as indicated by the increase in γH2AX (Figure 5A). In contrast to whole-cell lysates, treatment with CPT, HU, and IR resulted in altered CPC protein abundance in the different sub-cellular fractions. INCENP was upregulated in the soluble nuclear fraction after treatment with CPT, and in the chromatin-bound fraction after IR. In particular, Survivin levels were substantially elevated in the chromatin-bound fraction after treatment with DNA-damaging agents, resembling the situation observed for Aurora B kinase. These data again underline our hypothesis that the CPC is functionally involved in the response to replication stress.

Unresolved replication stress manifests in various forms, such as under-replicated sites, metaphase breaks, anaphase bridges, and 53BP1 nuclear bodies [34]. Consequently, we investigated the effect of Survivin depletion on the coping mechanisms of damage-induced replication stress. First, we focused on the mitotic DNA synthesis (MiDAS) [35]. Here, the replication machinery is unable to complete replication by the end of G2; therefore, the cells continue to replicate during mitosis. To investigate whether the depletion of CPC members results in delayed genome replication during mitosis, 5-ethynyl-2′-deoxyuridine (EdU) incorporation assays were performed. In addition to Survivin- or Aurora B depletion, the cells were incubated with the CDK1 inhibitor RO-3306 to arrest them in the G_2_ phase. Indeed, the depletion of Aurora B and Survivin resulted in an increase in mitotic EdU foci. However, this effect was much more pronounced and significant for Survivin (Figure 5B). As mitotic DNA synthesis often generates breaks in metaphase chromosomes, we performed chromosome spreading after Survivin depletion. In brief terms, after 48 h of depletion, the cells were incubated with 0.08 μg/mL of colcemide for 2 h to arrest them in metaphase and spread the chromosomes on microscopic slides. Survivin-depleted cells revealed a significant increase in breaks per metaphase, and the number of metaphases with breaks was increased (Figure 5C). Notably, DNA lesions that arise during mitosis induce the formation of 53BP1 nuclear bodies (NBs) at loci that fail to complete DNA replication. These lesions are subsequently sequestered into large chromatin domains that are enriched in 53BP1 and other DDR markers to promote repair after mitosis. Indeed, the depletion of Survivin with different targeting siRNAs significantly increased the formation of 53BP1 NBs per cell nucleus (Figure 5D,E). In summary, these data functionally mark the CPC protein Survivin as a pivotal resistor against replication stress.

### 3.7. The CPC Is Involved in Translesion Synthesis

In case cells fail to directly repair DNA lesions, the mechanisms of damage tolerance have evolved as elements of genomic maintenance programs to resolve replication stress [10]. One of these highly conserved DDT pathways is translesion synthesis (TLS), which allows the DNA replication machinery to replicate past DNA lesions and prevent the collapse of stalling forks. TLS involves switching out regular DNA polymerases for specialized translesion polymerases that can facilitate the insertion of bases opposite damaged nucleotides. Although this is error-prone and potentially leads to mutations, the priority during mitosis is to complete DNA replication and ensure proper chromosome segregation. Notably, polymerase switching is thought to be mediated via post-translational modifications of the replication processivity factor PCNA, among other factors. Polymerases, such as Pol η, Pol ζ, and REV1, are recruited to the stalled replication fork and replicate past DNA lesions. Subsequently, the normal replicative DNA polymerase δ is restored, and DNA synthesis continues.

**Figure 6:**
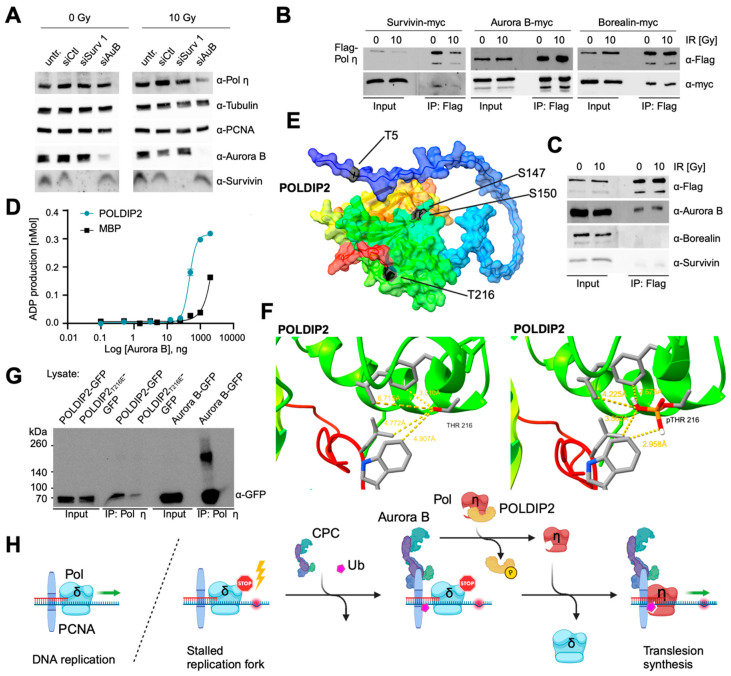
POLDIP2 is a substrate of Aurora B kinase, and it links the CPC to translesion synthesis. (**A**) Expression of polymerase η after depletion of CPC members and concurrent irradiation. HeLa cells were transfected with non-targeting, Survivin-specific, or Aurora B-specific siRNA and irradiated with 10 Gy 48 h later. Whole-cell extracts were generated and subjected to immunoblot analysis with indicated antibodies. Tubulin served as a loading control. (**B**,**C**) Co-immunoprecipitation reveals an interaction between CPC members and polymerase η. 293T cells were co-transfected with plasmids coding for FLAG-Pol η, together with the indicated myc-tagged CPC members. Then, 24 h later, cells were irradiated with 10 Gy and incubated for 2 h. Prior to immunoprecipitation, chromatin extraction was performed. FLAG-Pol η was immunoprecipitated from the extracts, and analysis was performed with the indicated antibodies. (**D**) POLDIP2 is a substrate of Aurora B kinase. Aurora B kinase assay performed with the known substrate MBP and POLDIP2. ADP production mirrors enzymatic activity. Both data sets have been fit with sigmoidal curves (POLDIP2: R2 = 0.99; MBP: R2 = 0.98). (**E**) Three-dimensional model of POLDIP2 (Q9Y2S7) from the AF2 database. ATR phosphorylation sites (S147, S150) are labeled, as well as possible Aurora B phosphorylation sites (T5, T216) according to PhosphoPICK. (**F**) Detail image of residues in close proximity of T216 (left). Several hydrophobic residues are located in the surrounding of T216 facing directly towards this residue. Following the phosphorylation of T216, distances to hydrophobic amino acids decrease (right). (**G**) Co-immunoprecipitation reveals a decreased interaction between POLDIP2 T216E and Pol η. 293T cells were transfected with plasmids encoding POLDIP2 wildtype-, T216E-, or AuroraB-GFP. Lysates were immunoprecipitated (IP) with Pol η-specific antibodies and immunoblotted for GFP. (**H**) Model of the CPC’s role in translesion synthesis. Upon DNA damage, PCNA is ubiquitinated at stalled replication forks and the CPC binds to PCNA via INCENP’s PIP-box motif. Sequestered Pol η is liberated after phosphorylation of POLDIP2 via Aurora B kinase. Due to the high affinity and close proximity, Pol η displaces Pol δ and resumes replication via translesion synthesis.

As we have already demonstrated that the CPC interacts with PCNA and functionally supports faithful DNA replication and fork progression, it was logical to elucidate a possible role in TLS. As such, the expression of Pol η was analyzed in Survivin- and Aurora B kinase-depleted cells dependent on IR. Immunoblot analysis revealed that Pol η expression was decreased, particularly after the depletion of Aurora B in combination with IR and, to a lesser extent, after Survivin depletion (Figure 6A). Notably, PCNA protein levels were unaffected by depletion or IR. Indeed, co-immunoprecipitation on chromatin extracts with FLAG-Pol η revealed an interaction with the myc-tagged CPC members Survivin, Aurora B, and Borealin (Figure 6B). Moreover, we verified these results for endogenous Survivin and Aurora B (Figure 6C). However, we were unable to detect endogenous Borealin in the eluates.

Importantly, Pol η is sequestered by the POLDIP2 under normal conditions [36]. POLDIP2 exhibits a very high affinity for mono-ubiquitinated PCNA, a modification to mark sites of stalled replication forks. The release of Pol η from POLDIP2 depends on phosphorylation events, such as at S147 and S150 via the kinase Ataxia telangiectasia and Rad3-related (ATR) [36]. As such, it was tempting to speculate that Aurora B could also phosphorylate POLDIP2, and we consequently performed an assay to measure kinase activity based on ADP production (Figure S12). The known Aurora B substrate MBP [37] was included as a positive control, and both substrates were utilized at the same concentration, together with increasing kinase concentrations (0–200 ng). Indeed, POLDIP2 was identified as a substrate of Aurora B at concentrations above 50 ng (Figure 6D). Notably, the phosphorylation of the known substrate MBP required higher concentrations of Aurora B, indicative of a possibly higher affinity for POLDIP2. Next, we performed a bioinformatic screen for possible phosphorylation motifs that have been reported to be variable for Aurora B kinase (R/K-X1-3-S/T) [38]. Briefly, the sequence of human POLDIP2 was obtained from UniProt (Q9Y2S7) and analyzed using PhosphoPICK [39]. Two possible phosphorylation sites were identified at T216 and T5 (Table S1). As the site scores were not very high, possible phosphorylation sites (ARET^216^LRA; AACT^5^ARR) were additionally analyzed via PPSP (prediction of PK-specific phosphorylation site), where the motif surrounding T216 showed higher scores (percentile > 90; score: 1.88). Indeed, T216 is located on an exposed α-helical stretch [36], and a model of POLDIP2 predicted via AF2 (model: Q9Y2S) [40] revealed that S147, S150, and T216 are located on the same side of POLDIP2, facing in the same direction (Figure 6E). The distances measured from the model indicate that the closest distance from the next hydrophobic residue (F200) to the oxygen atom of T216 is 3.3 Å, which is close enough to be within the range of London-dispersion forces (Figure 6F, left). Via the phosphorylation of T216, the surrounding hydrophobic residues are exposed to polarized oxygens of the phosphate group 0.7 Å to 1.9 Å closer than before (Figure 6F, right), which might indeed favor the subsequent release of Pol η. To confirm our hypothesis, we introduced a phospho-mimicking T216E mutation to POLDIP2. Co-immunoprecipitation experiments (Figure S13) revealed that Pol η binding was severely limited in the phospho-mimicry setting compared to the POLDIP2 wildtype or Aurora B (Figure 6G). This confirmed that T216 of POLDIP2 is a phosphorylation target for the release of Pol η.

## 4. Discussion

The chromosomal passenger complex (CPC), primarily studied in mitosis, also plays important roles during interphase, though its localization and function in this phase are less understood. Studies indicate that histone H3Ser10 phosphorylation via Aurora B occurs before mitosis, suggesting CPC–chromatin interaction during interphase. Specifically, the CPC is linked with pericentric heterochromatin in the late S phase through INCENP/HP1 interaction, and various interphase phosphorylation targets of Aurora B, including p53 and p21, affect CDK1 activation. The CPC members, Survivin, Borealin, and INCENP, are present during interphase, and Survivin’s upregulation in cancer suggests a role beyond its mitotic functions. Survivin is known for its anti-apoptotic role and its deregulation in cancer due to mechanisms such as gene amplification or signaling-pathway alterations (PI3K and MAPK). Survivin’s expression in cancer cells is often independent of the cell cycle, contributing to tumor resistance against apoptosis. During interphase, Survivin predominantly localizes in the cytoplasm, but its inhibition of interaction with CRM1 leads to nuclear accumulation. The degradation of Survivin and other CPC members via APC/C Cdh1 from late mitosis to G1 phase suggests their importance in S phase. Evidence links Survivin to the G1/S transition, enhancing the S and G2/M phases in cancer cells. Moreover, Survivin upregulation due to growth factors and cytokines supports hematopoietic progenitor maturation and the G0/G1-to-S-phase transition. Recent studies also implicate Survivin in the DNA damage response (DDR). For instance, Survivin is involved in radiation resistance and DNA repair in cancer cells, suggesting that it interacts with the DNA repair machinery. Our study revealed that irradiation induces Survivin relocation from the cytoplasm to the nucleus and formation of nuclear foci in A431 cells, confirmed in p53-wildtype HeLa and U2OS cells. The presence of CPC components (Aurora B, Borealin, and INCENP) in these foci post-irradiation indicates the complex’s role in DDR. During mitosis, Survivin binds to a histone mark (H3Thr3p) to localize CPC to chromosomes, while Borealin, phosphorylated via cyclinB-CDK1, aids in CPC centromeric localization. Aurora B, activated via INCENP, prevents premature chromosome segregation through phosphorylating kinetochore proteins. CPC’s centromeric localization in DDR highlights its involvement in repair processes, as centromeric regions contain repetitive DNA that hinders repair. We observed that irradiation-induced CPC foci accumulate at centromeric heterochromatin, confirmed via HP1α markers. Notably, non-CPC kinetochore proteins did not show similar nuclear foci after irradiation, implying a unique role for CPC in centromeric DNA repair. DNA lesions in heterochromatin require chromatin remodeling for efficient repair, often involving the dissociation of HP1 from H3K9me via Aurora B-mediated phosphorylation. Survivin depletion increases H2AX phosphorylation, indicating higher DNA damage levels, and nuclear foci formed via DDR proteins, like γH2AX and pATM, are absent from Survivin foci, suggesting an indirect role of Survivin in DDR. Most likely, a mixture of single- and double-stand breaks is induced: After Survivin depletion, major chromosome breaks are detectable as a consequence of mitotic DNA synthesis (MiDAS), representing DSBs. This perfectly makes sense, as stressed forks (which develop as a direct consequence of Survivin depletion) are managed through rescue from adjacent forks, repriming, translesion synthesis, template switching, and fork reversal, which produces a single-ended double-strand break (seDSB). Stressed forks, however, also collapse to seDSBs when they encounter single-strand nicks or are cleaved due to structure-specific nucleases. Regarding the role of the CPC in replication, we identified a possible interaction between INCENP and PCNA, a key replication factor, through a PIP-box motif. This suggests that CPC may support DNA replication, with INCENP mediating CPC’s proximity to PCNA. We demonstrated that CPC co-localizes with PCNA, particularly in the late S phase, and PLA analysis revealed close proximity between CPC components and PCNA. Survivin depletion decreased the replication-fork velocity, implicating CPC in maintaining replication speed. As replication forks stall under stress, CPC may aid replication-fork stability, preventing collapse and DNA damage. CPC depletion, particularly that of Survivin, led to increased gaps and breaks in metaphase chromosomes, signaling replication stress. Survivin-depleted cells also exhibited more 53BP1 nuclear bodies, indicating incomplete replication. Replication stress results in delayed genome replication, marked by EdU foci in mitotic cells. CPC might promote replication bypass mechanisms, such as translesion synthesis (TLS), where PCNA recruits error-prone polymerases to bypass DNA lesions. We observed a CPC association with the translesion polymerase Pol η, with Pol η expression decreasing after Aurora B depletion. Aurora B phosphorylates POLDIP2, a chaperone regulating Pol η’s access to replication forks. Our kinase assays confirmed that Aurora B phosphorylates POLDIP2 at T216, modulating Pol η’s interaction with PCNA. A T216E mutant of POLDIP2 showed decreased Pol η binding, suggesting that CPC, through Aurora B-mediated phosphorylation, regulates polymerase exchange at stalled replication forks. Thus, a CPC-facilitated polymerase switch could constitute an urgently required support mechanism, particularly in susceptible DNA regions like centromeric regions that are particularly prone to damage. Here, the CPC may function as an adaptor that recruits translesion polymerases to sites of stalled replication forks and mediates the contact between PCNA in the stalled replisome and the recruited TLS polymerases. In summary, we hypothesize that, under normal conditions, the CPC is not associated with proteins of the replication machinery and that Pol η is sequestered due to POLDIP2 (Figure 6H). Following IR, the CPC accumulates at the sites of DNA lesions via INCENP’s PIP-box motif. In addition to phosphorylation via ATR, Aurora B is also able to liberate POLDIP2 from Pol η, which, in turn, replaces Pol ∂ to resume the replication of damaged sites through translesion synthesis.

## 5. Conclusions

Our findings reveal the crucial role of Survivin and the Chromosomal Passenger Complex (CPC) in the response to DNA damage and replication stress, underscoring its importance in maintaining genomic integrity. Survivin and the CPC are present in heterochromatic foci in response to radiation, and the depletion of Survivin provokes replication stress and a reduction in fork speed. As such, the CPC coordinates replication and repair processes, ensuring replication fork stability and efficient DNA lesion repair. During replication, the CPC is associated with PCNA via a PIP-box motif identified in INCENP. Moreover, Survivin depletion leads to mitotic DNA synthesis (MiDAS) and chromosome breakage. Notably, the CPC facilitates translesion synthesis by recruiting non-classical DNA polymerases, such as DNA Pol η, to stalled replication forks in damaged DNA in centromeric regions. Here, we identified Aurora B as the kinase to phosphorylate POLDIP2 and resume replication via Pol η/translesion synthesis. And, last but not least, this study highlights the pervasive overexpression of Survivin in tumors, which enhances resistance to radio- and chemotherapy, beyond its role in mitosis.

## Data Availability

All data generated or analyzed during this study are included in this published article and its supplementary information files.

## Supplementary Materials

The following supporting information can be downloaded at https://www.mdpi.com/article/10.3390/cells13211804/s1: Figure S1: Irradiation-induced formation of Survivin nuclear foci. Figure S2: Kinetics of irradiation-induced Survivin expression and foci formation. Figure S3: Survivin accumulation in centromeric regions after irradiation. Figure S4: Localization of centromere and kinetochore components after irradiation. Figure S5: Distinct localization of Survivin foci and bona fide DNA repair proteins. Figure S6: Co-localization of CPC members with centromeric heterochromatin in S phase. Figure S7: Localization of Aurora B foci at centromeric heterochromatin during replication. Figure S8: Schematic illustration of the DNA fiber assay, colored tracks, and microscopic images of immunostained DNA fibers. Figure S9: Schematic illustration of the in situ proximity ligation assay (PLA). Figure S10: Sequence analysis revealing a conserved PIP-box motif in INCENP. Figure S11: The interaction between PCNA and INCENP PIP box is reduced by T2AA. Figure S12: Schematic illustration of the Aurora B kinase assay. Figure S13: Schematic illustration of the co-immunoprecipitation of Pol η and POLDIP2. Table S1: Results of the sequence analysis of POLDIP2 via PhosphoPICK yielding two possible Aurora B kinase phosphorylation sites. Reference [2] is cited in Supplementary Materials.
