# Supplementary material for: Old Passengers as New Drivers: Chromosomal Passenger Proteins Engage in Translesion Synthesis
Source: Cells. 2024 Oct 31;13(21):1804. doi: 10.3390/cells13211804 (PMC11544903; doi:10.3390/cells13211804)
Supplement: Supplementary file 1 [file cells-13-01804-s001.zip › cells-3268698-supplementary.pdf]

Old passengers as new drivers: chromosomal passenger proteins engage in translesion synthesis

Katharina Falke[1,‡] Elisabeth Schröder[1,‡] Stefanie Mosel[1,‡] Cansu N. Yürük[1], Sophie Feldmann[1], Désirée Gül[2], Paul Stahl[1], Roland H. Stauber[2] and Shirley K. Knauer[1,*]

[1]Institute for Molecular Biology II, Centre for Medical Biotechnology (ZMB), University of Duisburg-Essen, Universitätsstrasse 5, D-45141 Essen, Germany
[2]Molecular and Cellular Oncology, University Medical Center Mainz, Langenbeckstrasse 1, D-55101 Mainz, Germany

[‡]authors contributed equally
[*]Correspondence to:
Shirley K. Knauer
Institute for Molecular Biology II, Centre for Medical Biotechnology (ZMB), University of Duisburg-Essen, Universitätsstrasse 5, D-45141 Essen, Germany
Phone: +49-(0)201-183-4987
Fax: +49-(0)201-183-6053
E-mail: shirley.knauer@uni-due.de

Content

List of Supplementary Figures and Tables *2*

Supplementary Figures *4*

Supplementary Tables *19*

List of Supplementary Figures and Tables

Figure S1. Irradiation-induced formation of Survivin nuclear foci.

Figure S2. Kinetics of irradiation-induced Survivin expression and foci formation.

Figure S3. Survivin accumulation in centromeric regions after irradiation.

Figure S4. Localization of centromere and kinetochore components after irradiation.

Figure S5. Distinct localization of Survivin foci and bona fide DNA repair proteins.

Figure S6. Co-localization of CPC members with centromeric heterochromatin in S phase.

Figure S7. Localization of Aurora B foci at centromeric heterochromatin during replication.

Figure S8. Schematic illustration of the DNA fiber assay, colored tracks, and microscopic images of immunostained DNA fibers.

Figure S9. Schematic illustration of the *in situ* Proximity Ligation Assay (PLA).

Figure S10. Sequence analysis revealing a conserved PIP box motif in INCENP.

Figure S11. The interaction between PCNA and INCENP PIP box is reduced by T2AA.

Figure S12. Schematic illustration of the Aurora B kinase assay.

Figure S13. Schematic illustration of the co-immunoprecipitation of Pol η and POLDIP2.

Table S1. Results of the sequence analysis of POLDIP2 by PhosphoPICK yielding two possible Aurora B kinase phosphorylation sites.

Supplementary Figures

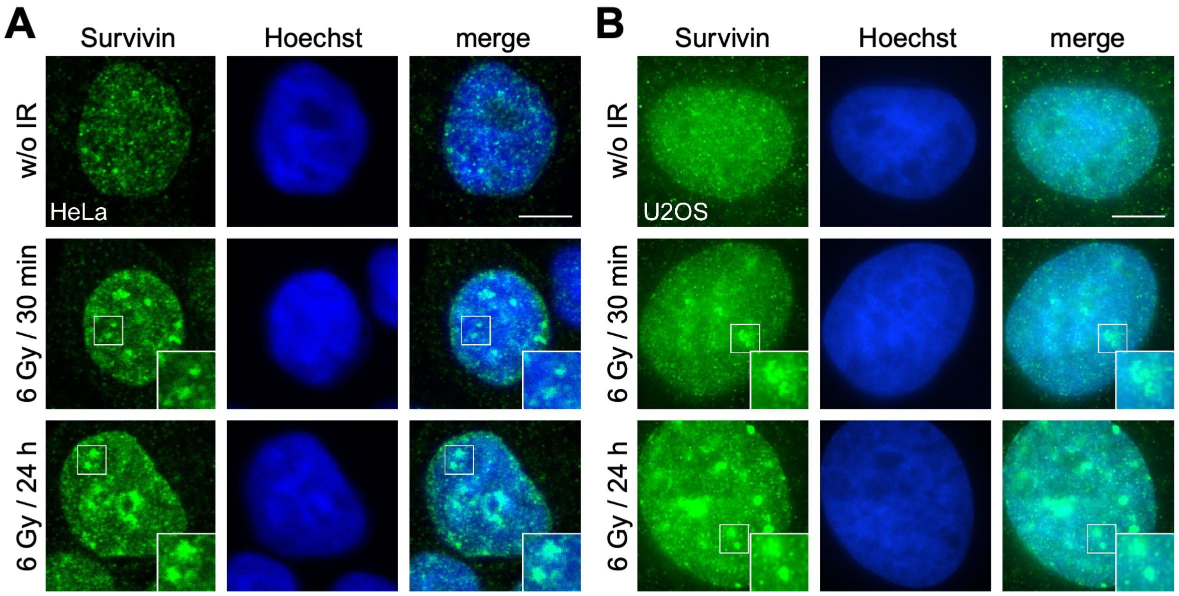

Figure S1. Irradiation-induced formation of Survivin nuclear foci. A./B. Parental HeLa (**A**) and U2OS (**B**) cells were irradiated with 6 Gy, fixed 30 min and 24 h after IR and permeabilized. Cells were immunostained with a Survivin-specific antibody (green) for confocal microscopy. DNA was stained with Hoechst (blue). Non-irradiated cells (w/o IR) were used as a control. Scale bar, 7.5 µm. The insets show higher magnifications of the areas outlined in the main panels.

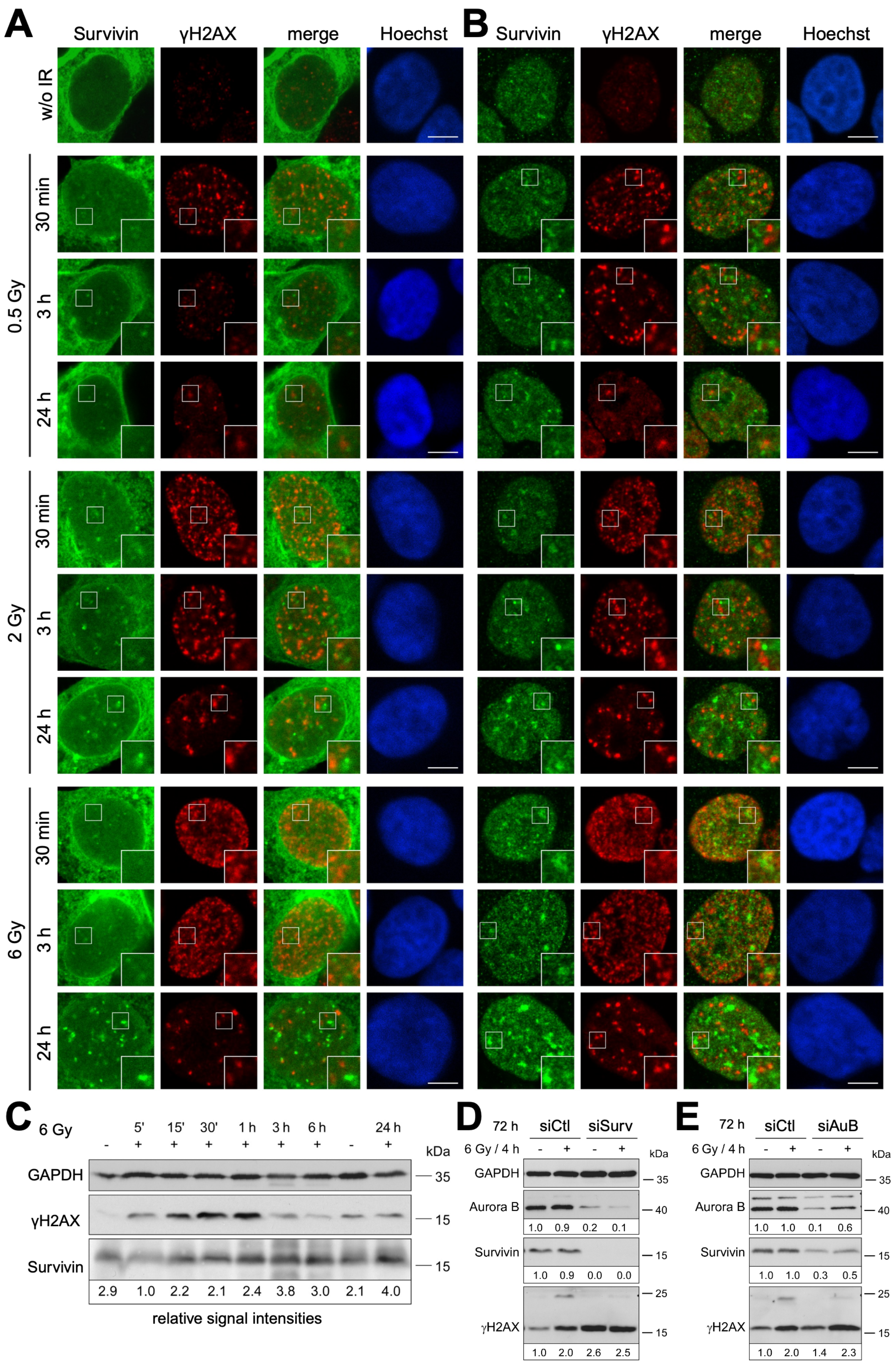
A
Survivin
γH2AX
merge
Hoechst
B
Survivin
γH2AX
merge
Hoechst
w/o IR
30 min
3 h
24 h
0.5 Gy
2 Gy
6 Gy
C
6 Gy
5'
15'
30'
1 h
3 h
6 h
24 h
- + + + + + + - +
kDa
GAPDH
γH2AX
Survivin
35
15
15
2.9 1.0 2.2 2.1 2.4 3.8 3.0 2.1 4.0
relative signal intensities
D
72 h
siCtl
siSurv
6 Gy / 4 h
- + - +
kDa
GAPDH
Aurora B
1.0 0.9 0.2 0.1
Survivin
1.0 0.9 0.0 0.0
γH2AX
1.0 2.0 2.6 2.5
35
40
15
25
15
E
72 h
siCtl
siAuB
6 Gy / 4 h
- + - +
kDa
GAPDH
Aurora B
1.0 1.0 0.1 0.6
Survivin
1.0 1.0 0.3 0.5
γH2AX
1.0 2.0 1.4 2.3
35
40
15
25
15

Figure S2. Kinetics of irradiation-induced Survivin expression and foci formation. **A/B.** A431 cells stably expressing Survivin-GFP (**A**) as well as parental A431 cells (**B**) were irradiated with 0.5, 2 and 6 Gy, respectively, fixed 30', 3 h and 24 h after IR and permeabilized. Cells were immunostained with a γH2AX (pSer137)-specific antibody (red) for confocal microscopy. Cells in **B** were additionally stained with a Survivin-specific antibody (green). DNA was stained with Hoechst (blue). Non-irradiated cells (w/o IR) were used as a control. Scale bar, 7.5 µm. The insets show higher magnifications of the areas outlined in the main panels. **C**. Kinetics of irradiation-induced Survivin expression. A431 cells stably expressing Survivin-GFP were irradiated with 6 Gy, and whole-cell extracts were prepared at the indicated time points after IR for immunoblot analysis using specific antibodies as indicated. The relative signal intensities of Survivin bands were quantified by densitometric analysis using ImageJ and normalized to GAPDH. The depicted values represent the relative increase in Survivin levels after IR. **D./E.** Immunoblot analysis of γH2AX levels in Survivin- (**D**) and Aurora B- (**E**) depleted cells. Parental A431 cells were transfected siRNA specific for Survivin (siSurv, **D**) or Aurora B (siAuB, **E**) or with 20 nM non-targeting control siRNA (siCtl) and irradiated with 6 Gy 68 h after transfection. Whole-cell extracts were prepared 4 h after IR for immunoblot analysis using specific antibodies as indicated. Relative signal intensities of the Aurora B, Survivin and γH2AX bands were quantified by densitometric analysis using ImageJ and normalized to GAPDH. The depicted values represent the relative decrease/increase in protein levels after knock-down/irradiation.

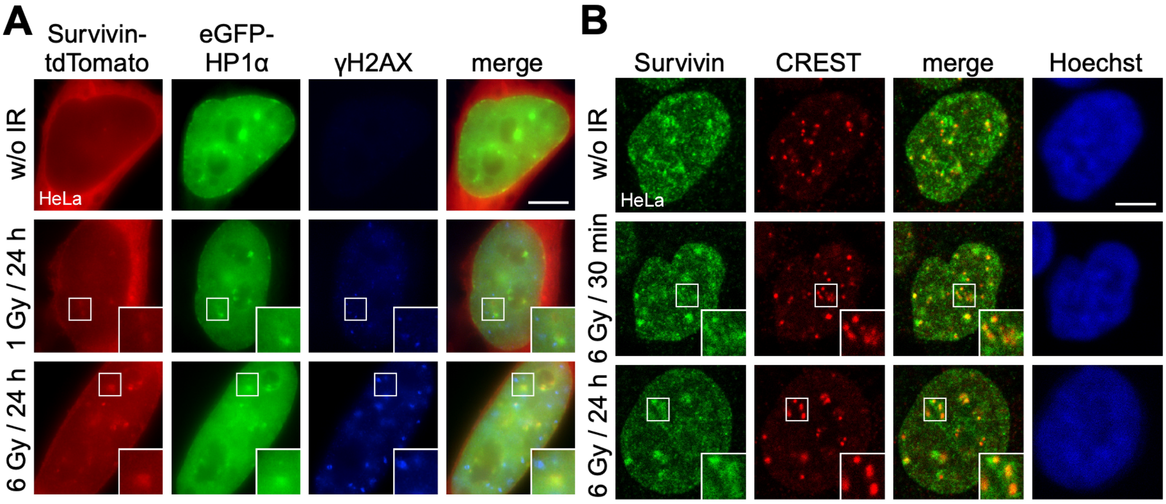

Figure S3. Survivin accumulation in centromeric regions after irradiation. A. HeLa cells were co-transfected with plasmids coding for Survivin-tdTomato and eGPF-HP1α. Cells were irradiated with 1 and 6 Gy 24 h after transfection, fixed 24 h after IR, immunostained with a γH2AX-specific antibody (blue) for fluorescence microscopy. **B.** Parental HeLa cells were irradiated with 6 Gy, fixed 30 min and 24 h after IR and permeabilized. Cells were immunostained for endogenous Survivin (green) and with CREST serum detecting centromeric chromatin (red) for confocal microscopy. DNA was stained with Hoechst (blue). Non-irradiated cells (w/o IR) were used as a control. Scale bar, 7.5 µm. The insets show higher magnifications of the areas outlined in the main panels.

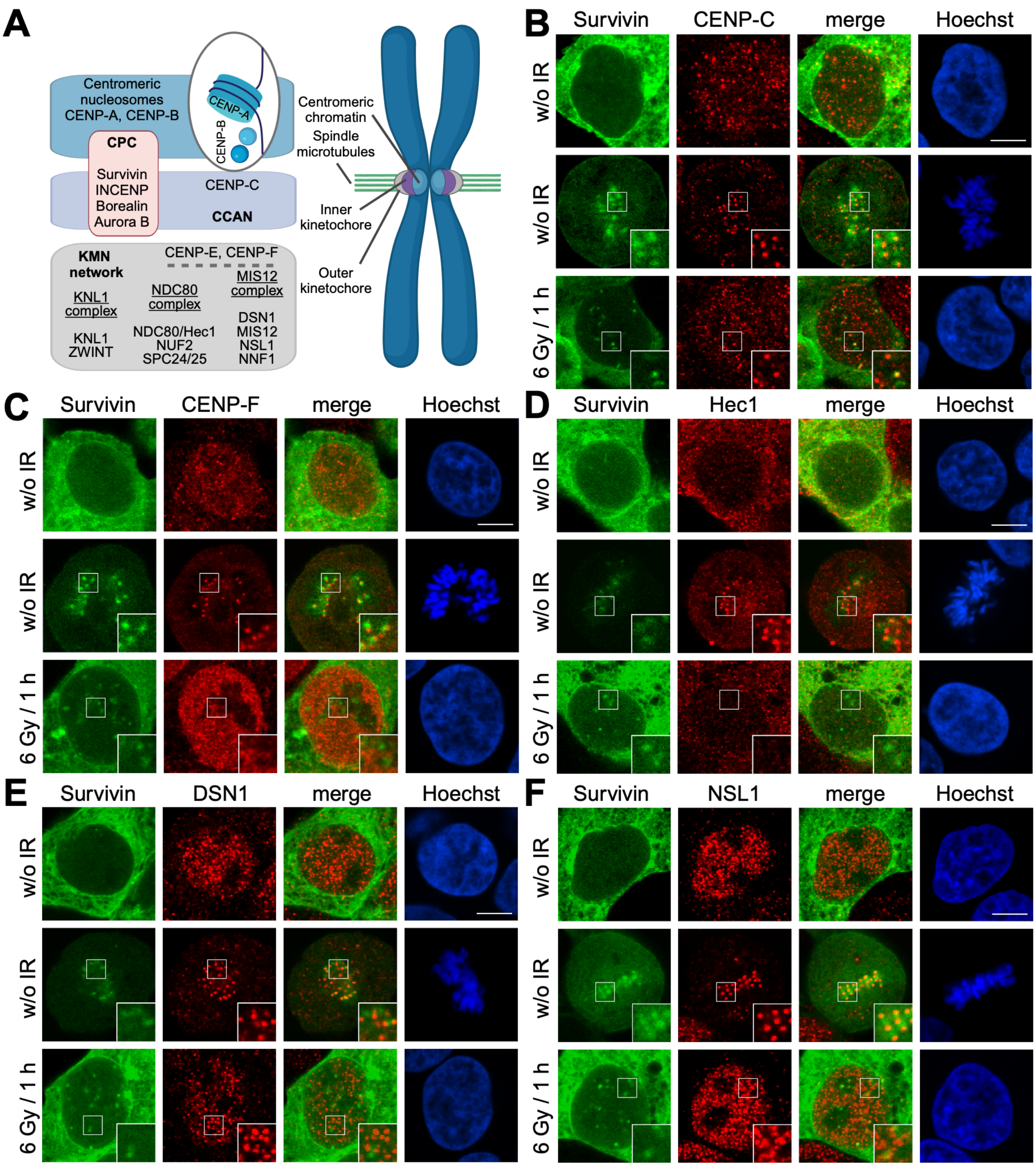

Figure S4. Localization of centromere and kinetochore components after irradiation. A. Schematic overview on the components constituting the centromere and the different kinetochore regions. **B.-F.** A431 cells stably expressing Survivin-GFP were irradiated with 6 Gy and fixed 1 h after irradiation. Cells were immunostained with antibodies specific for CENP-C (**B**), CENP-F (**C**), Hec1 (**D**), DSN1 (**E**), and NSL1 (**F**) (red) for confocal microscopy. DNA was stained with Hoechst (blue). Non-irradiated cells (w/o IR) were used as a control, and representative images of mitotic cells are shown to indicate specific antibody binding. Scale bar, 7.5 µm. The insets show higher magnifications of the areas outlined in the main panels.

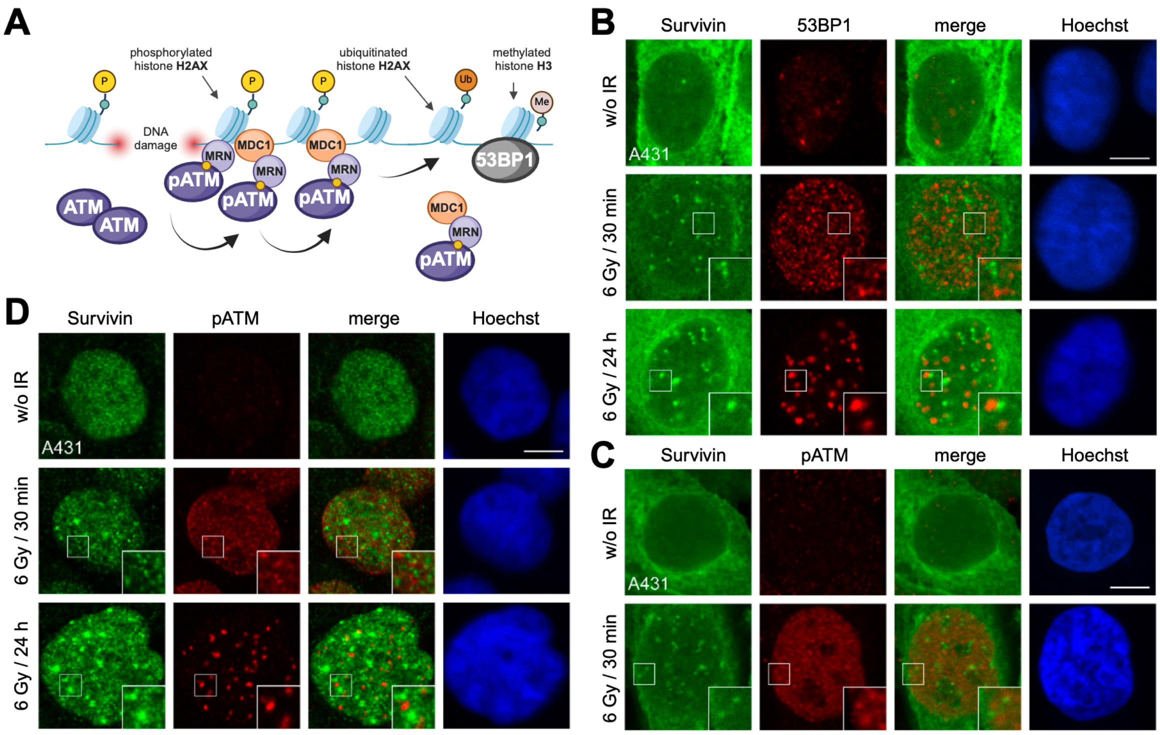

Figure S5. Distinct localization of Survivin foci and *bona fide* DNA repair proteins. A. The DNA damage response cascade. The first step in the DDR activation is the recruitment of ATM kinase by the MRN complex to DSBs. At the DSB, ATM is activated and phosphorylates both itself and other substrates, including H2AX. γH2AX is recognized and bound by MDC1 that then enables further accumulation of ATM-MRN complex and spreading of γH2AX around DSBs. ATM-mediated phosphorylation of MDC1 allows also for the recruitment of a ubiquitin ligases. Ubiquitination of H2A together with the deposition of methyl group on histone H3 results in the recruitment of 53BP1, which further coordinates DNA repair pathway choice. **B.-D.** A431 cells stably expressing Survivin-GFP (**B**/**C**), as well as parental A431 cells (**D**) were irradiated with 6 Gy, fixed 30 min (**A**-**F**) and 24 h (**B** and **D**) after irradiation and permeabilized. Cells were immunostained with antibodies specific for 53BP1 (**B**), pATM (pS1981) (**C**/**D**), respectively (red) for confocal microscopy. Cells in **D** were additionally immunostained with a Survivin-specific antibody (green). DNA was stained with Hoechst (blue). Non-irradiated cells (w/o IR) were used as a control. p, phosphorylated. Scale bar, 7.5 µm. The insets show higher magnifications of the areas outlined in the main panels.

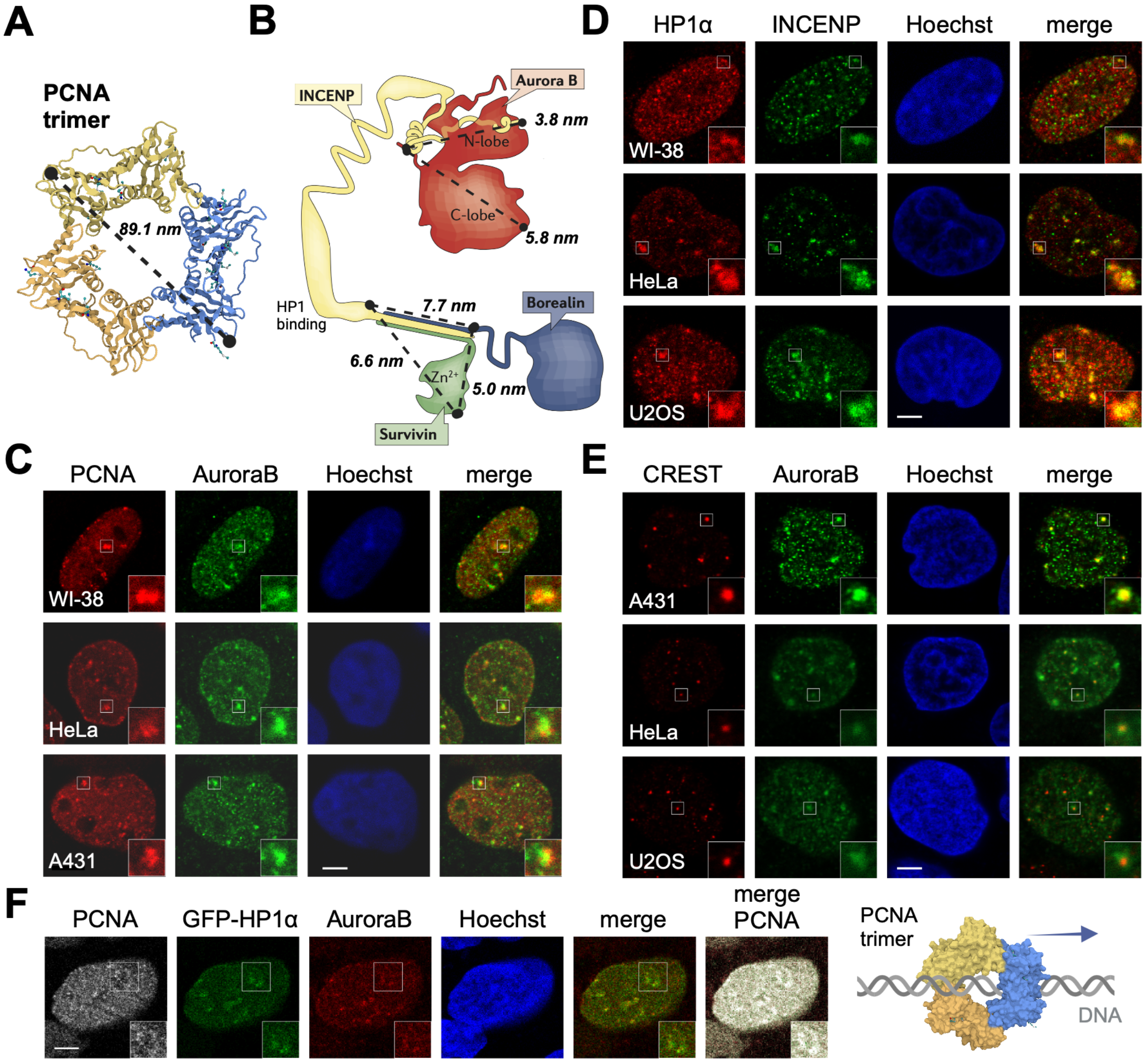
A
PCNA trimer
89.1 nm
B
INCENP
Aurora B
3.8 nm
N-lobe
C-lobe
5.8 nm
HP1 binding
7.7 nm
Borealin
6.6 nm
Zn2+
5.0 nm
Survivin
D
HP1α
INCENP
Hoechst
merge
WI-38
HeLa
U2OS
C
PCNA
AuroraB
Hoechst
merge
WI-38
HeLa
A431
E
CREST
AuroraB
Hoechst
merge
A431
HeLa
U2OS
F
PCNA
GFP-HP1α
AuroraB
Hoechst
merge
merge PCNA
PCNA trimer
DNA

Figure S6. Co-localization of CPC members with centromeric heterochromatin in S phase. A. The structure of the heterotrimeric PCNA sliding clamp reveals a total diameter of less than 90 nm (PDB code: 2HII). **B.** Schematic overview on the spatial organization of the CPC and the average distances between its members. With all interspaces ranging below 10 nm within the complex, indeed all CPC members might be in close proximity to the replication factor PCNA upon its direct binding to one factor. Modified from Carmena *et al*.[2] **C.** Aurora B co-localizes with PCNA. WI-38, HeLa and A431 cells were fixed, permeabilized and immunostained with antibodies specific for Aurora B- (green) and PCNA- (red) for confocal microscopy. **D.** INCENP co-localizes with HP1α. WI-38, HeLa and U2OS cells were fixed, permeabilized and immunostained with antibodies specific for HP1α- (red) and INCENP- (green) for confocal microscopy. **E.** Aurora B foci are located at centromeric heterochromatin. A431, HeLa and U2OS cells were fixed, permeabilized and immunostained with CREST centromere immune serum (red) and an Aurora B- (green) specific antibody for confocal microscopy. **F.** Aurora B and HP1α co-localize in S phase cells. U2OS cells were transfected with a plasmid coding for eGFP-HP1α (green). Cells were fixed 24 h after transfection, permeabilized and immunostained with PCNA- (white) and Aurora B- (red) specific antibodies for confocal microscopy. DNA was stained with Hoechst (blue). Scale bar, 5 µm. The insets show higher magnifications of the areas outlined in the main panels.

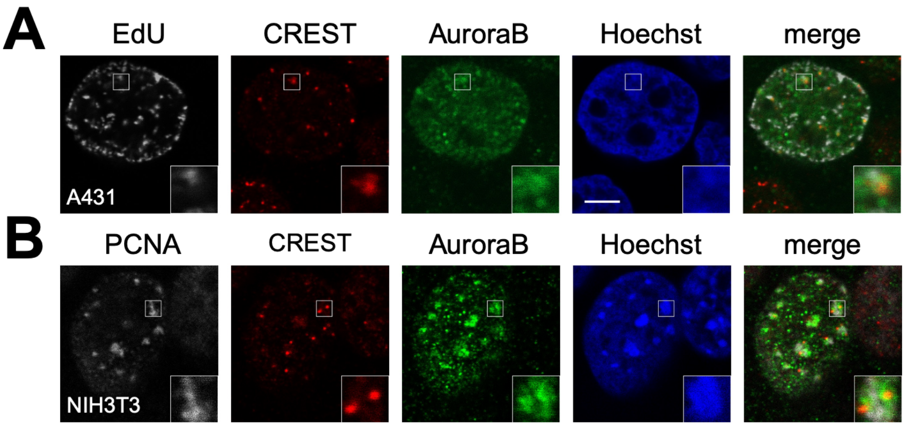

Figure S7. Localization of Aurora B foci at centromeric heterochromatin during replication. A. EdU (10 μM) was incorporated for 20 min before fixation to visualize replicating A431 cells (white). Cells were co-stained for Aurora B (green) and centromeres (CREST, red) for confocal microscopy. **B.** NIH3T3 cells were co-stained with PCNA- (white), Aurora B- (green) and centromere- (CREST, red) specific antibodies for confocal microscopy. DNA was stained with Hoechst (blue). Scale bar, 5 μm. The insets show higher magnifications of the areas outlined in the main panels.

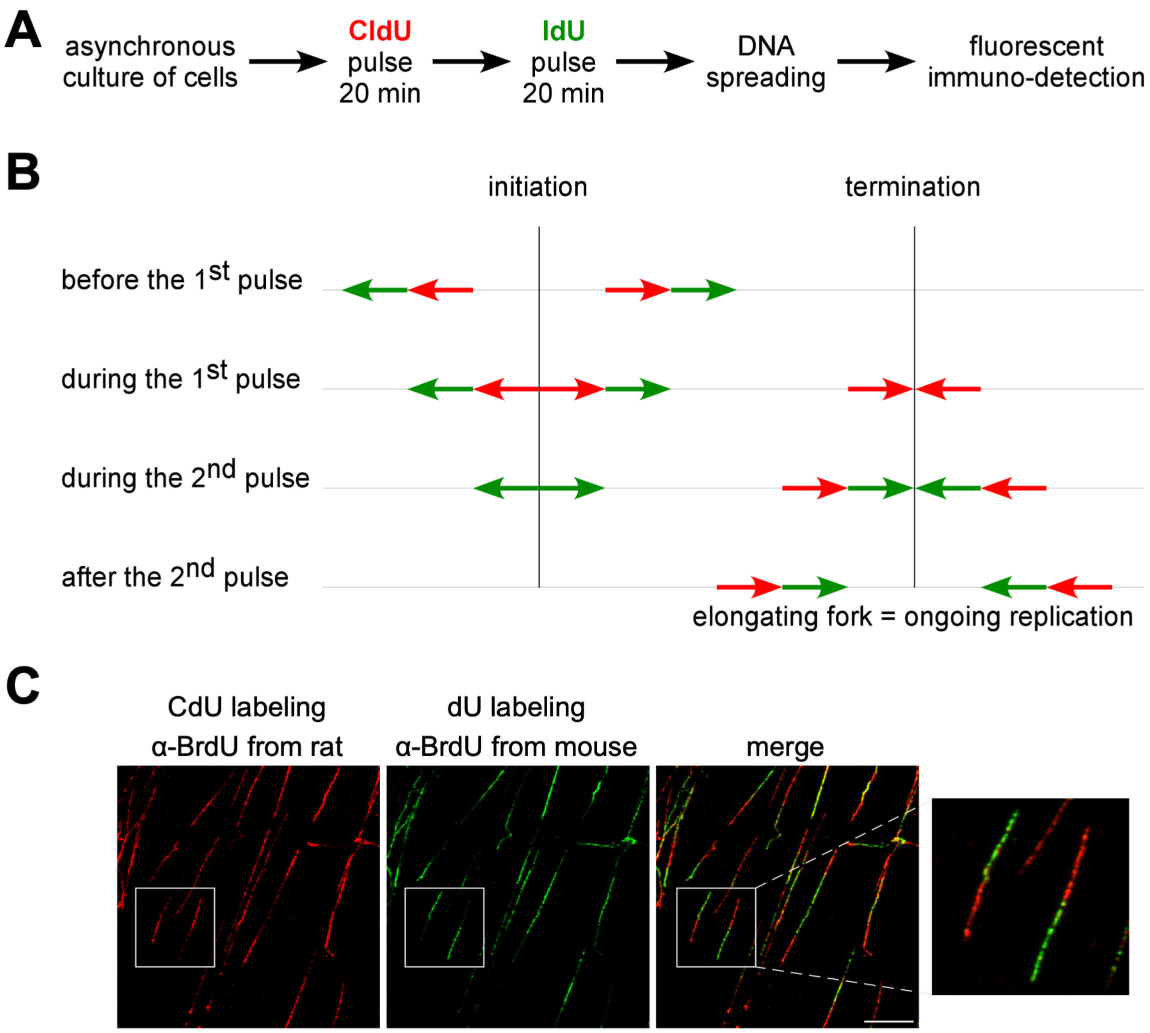

Figure S8. Schematic illustration of the DNA fiber assay, colored tracks, and microscopic images of immunostained DNA fibers. A. Workflow of the DNA fiber assay. **B.** Schematic representation of colored tracks to illustrate their meaning. Red-green tracks were classified as ongoing replication. **C.** Example of spread and immunostained DNA fibers from cells labeled with CldU and IdU for 20 min each. The fixed and denatured fiber spreads were stained with two different BrDU-specific antibodies. The right panel shows a higher magnification of the area outlined in the merge panel. Scale bar, 1 μm.

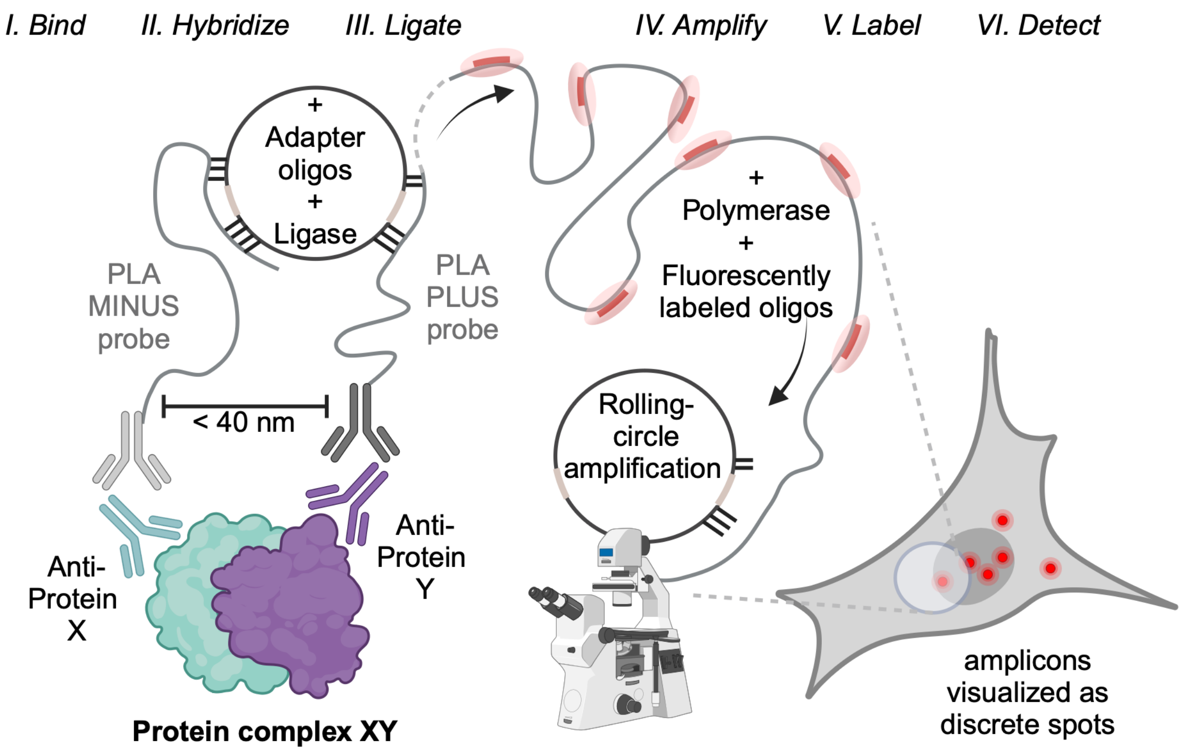

Figure S9. Schematic illustration of the *in situ* Proximity Ligation Assay (PLA). After pairs of primary antibodies have bound (I) a pair of interacting proteins X and Y (turquoise and purple) with distances below 40 nm, *in situ* PLA PLUS and MINUS probes conjugated to appropriate secondary antibodies are added. The conjugated oligonucleotides are joined and circularized by ligation upon introduction of hybridizing linear connector/adapter oligonucleotides (II, III). The resulting product serves as a template for rolling circle amplification fostered by a DNA polymerase (IV) in conjunction with fluorescently labeled oligonucleotides (V). Amplicons can be visualized as bright discrete spots inside the cells, allowing easy quantification after microscopic analysis.

A

APIM motif: (K/R)-(F/Y/W)-(L/I/V/A)-(L/I/V/A)-(K/R)

PIP box motif: Q-x-x-(L/I/M)-x-x-(F/Y)-(F/Y)

B

```
  1 MGTTAPGPIH LLELCDQKLM EFLCNMDNKD LVWLEEIQEE AERMFTREFS  50
 51 KEPELMPKTP SQKNRRKKRR ISYVQDENRD PIRRRLSRRK SRSSQLSSRR 100
101 LRSKDSVEKL ATVVGENGSV LRRVTRAAAA AAAATMALAA PSSPTPESPT 150
151 MLTKKPEDNH TQCQLVPVVE IGISERQNAE QHVTQLMSTE PLPRTLSPTP 200
201 ASATAPTSQG IPTSDEESTP KKSKARILES ITVSSLMATP QDPKGQGVGT 250
251 GRSASKLRIA QVSPGPRDSP AFPDSPWRER VLAPILPDNF STPTGSRTDS 300
301 QSVRHSPIAP SSPSPQVLAQ KYSLVAKQES VVRRASRRLA KKTAEEPAAS 350
351 GRIICHSYLE RLLNVEVPQK VGSEQKEPPE EAEPVAAAEP EVPENNGNNS 400
401 WPHNDTEIAN STPNPKPAAS SPETPSAGQQ EAKTDQADGP REPPQSARRK 450
451 RSYKQAVSEL DEEQHLEDEE LQPPRSKTPS SPCPASKVVR PLRTFLHTVQ 500
501 RNQMLMTPTS APRSVMKSFI KRNTPLRMDP KCSFVEKERQ RLENLRRKEE 550
551 AEQLRRQKVE EDKRRRLEEV KLKREERLRK VLQARERVEQ MKEEKKKQIE 600
601 QKFAQIDEKT EKAKEERLAE EKAKKKAAAK KMEEVEARRK QEEEARRLRW 650
651 LQQEEEERRH QELLQKKKEE EQERLRKAAE AKRLAEQREQ ERREQERREQ 700
701 ERREQERREQ ERREQERQLA EQERRREQER LQAERELQER EKALRLQKEQ 750
751 LQRELEEKKK KEEQQRLAER QLQEEQEKKA KEAAGASKAL NVTVDVQSPA 800
801 CTSYQMTPQG HRAPPKINPD NYGMDLNSDD STDDEAHPRK PIPTWARGTP 850
851 LSQAIIHQYY HPPNLLELFG TILPLDLEDI FKKSKPRYHK RTSSAVWNSP 900
901 PLQGARVPSS LAYSLKKH                                   918
```

C

		PIP box	
H. sapiens	853	QAIIHQYY	860
P. troglodytes	848	QAIIHQYY	855
M. musculus	817	QAIVHQYY	824
G. gallus	774	QAVIHQYY	781
X. tropicalis	834	QAIRQQYY	841
X. laevis	809	QAIRQQYY	816

D

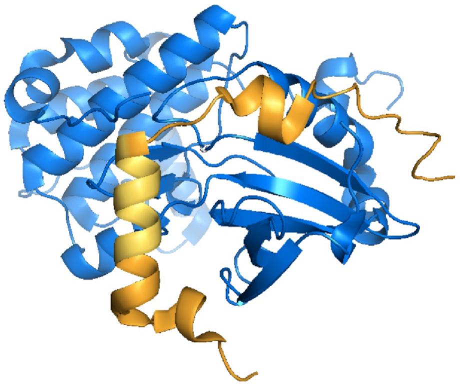

Figure S10. Sequence analysis revealing a conserved PIP box motif in INCENP. A. Consensus sequences of APIM (AlkB homolog 2 PCNA)- and PIP (PCNA-interacting protein)-binding motif. **B**. Amino acid sequence of human INCENP with the putative PIP box motif highlighted in yellow. **C.** Sequence alignments of the PIP box motif in INCENP homologs. Highly conserved residues are marked in yellow. Protein accession numbers: Homo sapiens: NP_001035784.1; Pan troglodytes: XP_001151913.1; Mus musculus: NP_057901.2; Gallus gallus: NP_990661.1; Xenopus tropicalis: NP_001121150.1; Xenopus laevis: NP_001081890.1. **D**. Crystal structure of Aurora B (aa 55-344; blue) in complex with INCENP (aa 835-903; orange) and its PIP box motif (aa 853-860; highlighted in yellow), PDB code: 4AF3.

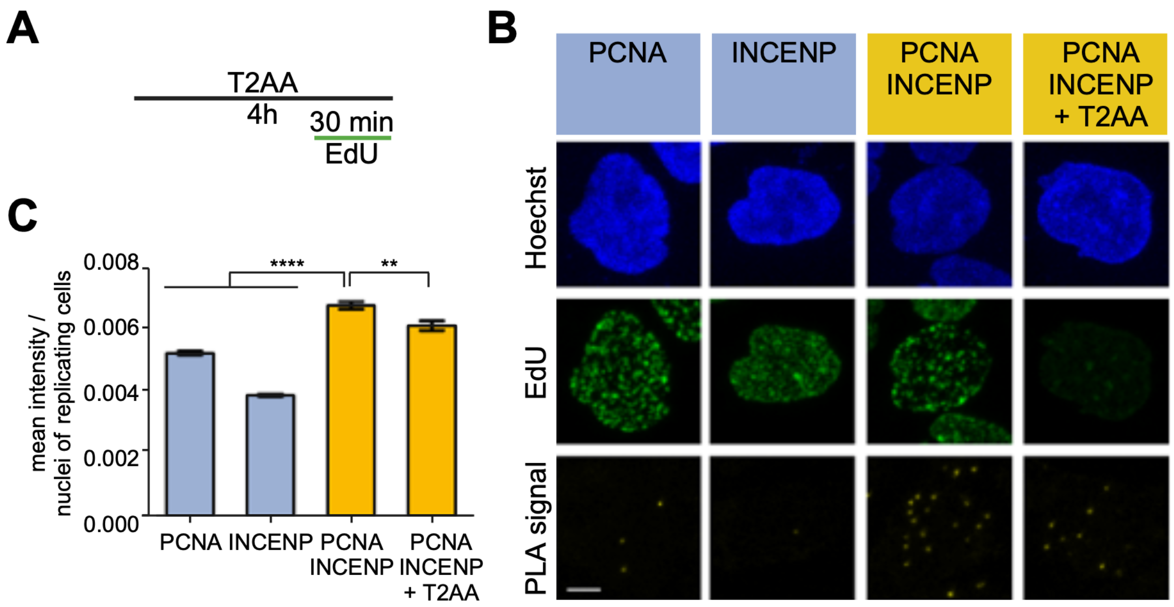

Figure S11. The interaction between PCNA and INCENP PIP box is reduced by T2AA. A. Schematic treatment workflow. HeLa cells were treated with 40 µM T2AA for 4 h. During the last 30 min of inhibitor treatment, EdU (10 µM) was to be incorporated in nascent DNA of replicating cells. **B.** Following treatment as described in **A**, EdU (green) was visualized via Click-iTTM Kit, and PLA was performed using primary antibodies against PCNA and INCENP. For negative controls, only a single primary antibody was used together with both PLA probes. Each yellow spot represents a single PLA interaction signal as detected by confocal microscopy. DNA was stained with Hoechst (blue). Scale bar, 5 µm. **C.** For quantification, the mean fluorescence intensity per nuclei of replicating cells was determined via Cell Profiler. Bar graphs represent means ± SEM of negative controls (light blue) and interaction samples (yellow).**, $p < 0.001$; ****, $p < 0.0001$.

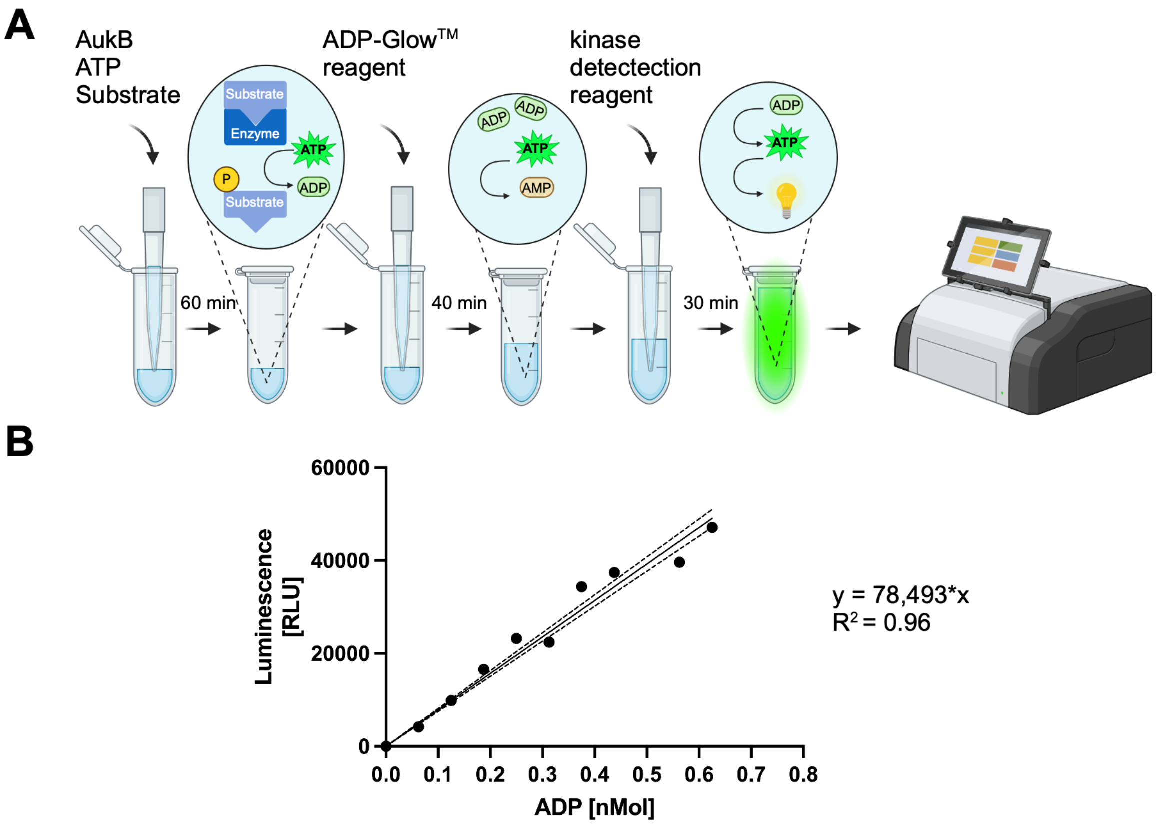

Figure S12. Schematic illustration of the Aurora B kinase assay. During the kinase reaction, ATP is converted to ADP to phosphorylate the substrate. In the following step, ADP-Glow^{TM} reagent is added which depletes the remaining ATP. Successively ADP is re-converted to ATP which is transformed into light by luciferase followed by plate reader analysis. **B.** ADP calibration curve correlating ADP content (nMol) and luminescent readout (RLU). Linear regression analysis in Prism9 (Graph Pad Software) resulted in a linear regression equation (R2=0.96) as visualized in the graph.

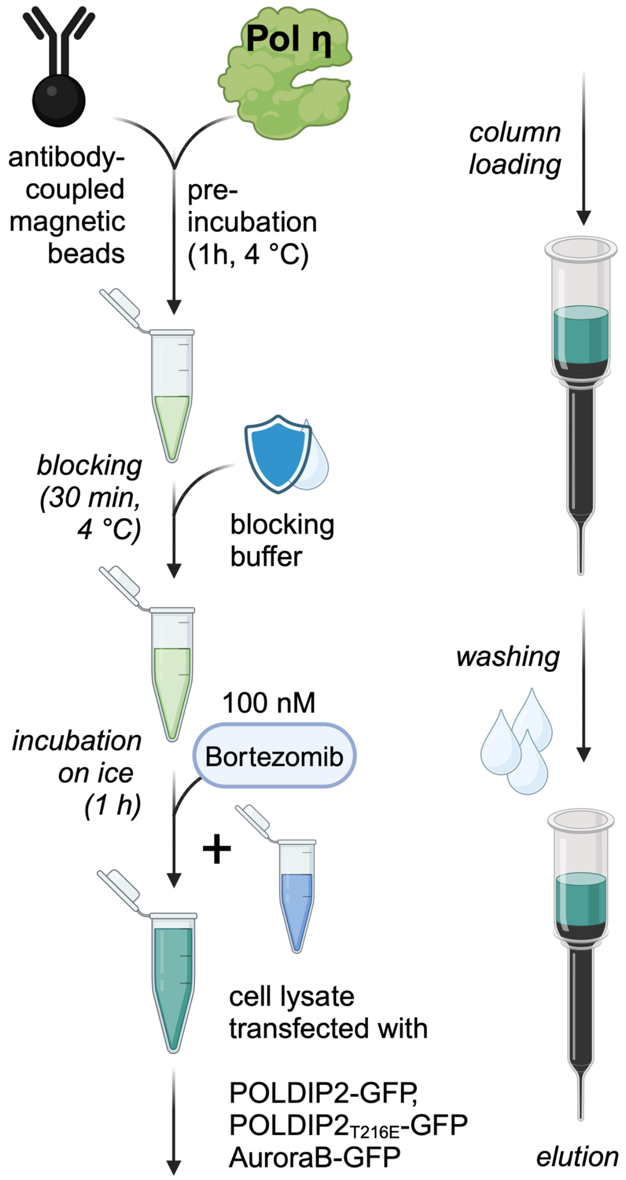

Figure S13. Schematic illustration of the co-immunoprecipitation of Pol η and POLDIP2. 293T cells were transfected with plasmids encoding POLDIP2-GFP, a $POLDIP2_{T218E}$-GFP mutant or Aurora B-GFP, and whole cell lysates were prepared 24 h after transfection. Magnetic beads covalently coated with anti-Pol η antibodies were pre-incubated with recombinant Pol η for 1 h at 4°C, and blocked for 30 min at 4°C. This was followed by addition of 100 nM bortezomib before incubation of the beads with the cell lysates on ice for 1 h. After loading the beads onto the column, bound proteins were eluted after several washing steps to be analyzed by immunoblotting.

Supplementary Tables

Table S1. Results of the sequence analysis of POLDIP2 by PhosphoPICK yielding two possible Aurora B kinase phosphorylation sites.

Identifier	Uniprot-Acc	blastp-identity	Kinase	Site	Site score	Site p-value
**Human POLDIP2**	Q9Y2S7	100.0	AURKB	216	0.734	0.008
**Human POLDIP2**	Q9Y2S7	100.0	AURKB	5	0.615	0.010
